# Initial Sugar Concentration on Sensory Characteristics of Raw Pu-Erh Tea Kombucha and Multi-Omics Analysis of the Fermentation Process Under Optimal Sugar Concentration

**DOI:** 10.3390/foods14183216

**Published:** 2025-09-16

**Authors:** Lingyun Yao, Hui Ma, Lingyang Yao, Haining Cao, Tao Feng, Huatian Wang, Chuang Yu, Min Sun

**Affiliations:** 1 School of Perfume and Aroma Technology, Shanghai Institute of Technology, Shanghai 201418, China; lyyao@sit.edu.cn (L.Y.); fengtao@sit.edu.cn (T.F.); sunmin@sit.edu.cn (M.S.); 2 Faculty of Materials Science and Chemistry, China University of Geosciences (Wuhan), Wuhan 430074, China; 3 Shandong Tianbo Food Ingredients Co., Ltd., Jining 272113, China

**Keywords:** kombucha, sensory evaluation, volatile compounds, metagenomic analysis, metabolomic analysis

## Abstract

The substrates of kombucha typically consist of tea and sugar. In this study, the effect of initial sugar concentration on volatile compound and sensory characteristics of raw Pu-erh tea (RAPT) kombucha was investigated. Compared to tea free (S1) and sugar free (S2) samples, the sugared tea (39 g/L sucrose in S3 and 78 g/L sucrose in S4) revealed better sensory quality and higher liking scores after the fermentation process. Hence, high-throughput sequencing analysis was performed to determine the variation in microbial composition between S3 and S4. The result showed that S4 exhibited higher abundances of *Komagataeibacter* and *Brettanomyces* as compared to S3. In addition, S4 presented the most favorable sensory qualities characterized by higher intensities of fruity, alcoholic, and fatty aromas, and the highest overall liking score. The metagenomic and metabolomic analysis was employed to further explore the metabolic pathways of RAPT kombucha under the optimal sugar concentration. The metagenomic and metabolomic analyses revealed that the pathways related to carbohydrate and amino acid metabolism were highly active under optimal sugar content, with compounds including glucose 6-phosphate, pyruvate and glutamate suggested to be important metabolites in regulating the sensory quality of the kombucha beverage. This paper provides a scientific basis for optimizing sugar addition in kombucha production.

## 1. Introduction

Kombucha is a traditional fermented drink that first appeared in northeastern China around 220 BCE and is now widely spread around the world [1]. The sugared tea (green tea or black tea) infusion is the most frequent used substrate for preparing traditional kombucha, which is fermented with symbiotic culture of bacteria and yeast (SCOBY) for several days to obtain the end products [2]. The consumption of tea kombucha has been linked to various health benefits, which are probably due to the bioactive compounds (arising from raw materials and enhanced by the fermentation process) and its role as a source of potentially prebiotic substances [3,4]. With the increasing demand for various functional and sensory characteristics, plenty of other plant materials such as fruit juices and herb infusions have been extensively used for kombucha fermentation in recent years [2,5].

The fundamental quality of tea-based kombucha is greatly attributed to its sensory features, which are commonly described as a sweet, slightly sour flavor, and carbonation [6,7]. These features are usually corelated with the number of factors during the fermentation process, such as the concentration of the fermentation substrate, oxygen supply, microbiological and technological parameters [7,8,9,10]. For example, a recent study has observed that the types of tea could significantly influence the quality of kombucha, with oolong tea and green tea kombucha exhibited higher overall sensory scores among the six main tea types in China [11]. As well as the component of tea leaves, sugar is also key substrate utilized in kombucha fermentation due to its crucial role on monitoring the growth of SCOBY and regulating the flavor quality [12,13]. Meanwhile, the taste of kombucha depends on the amount of residual sugar present in the mixture [7,14]. However, higher sugar concentration does not necessarily mean better quality characteristics. For instance, maqui juice-based kombucha exhibited the best response in quality parameters when added with 100 g/L of sugar content rather that higher concentrations (125 g/L and 150 g/L) [15]. Therefore, the concentration of sugar is of great importance for kombucha production process.

Typically, tea infusion is supplemented with 50 to 150 g/L of sugar for starting the kombucha fermentation process [6]. In a recent report, the initial sugar concentration has been proved to important processing condition that could affect the sensory profiles of black and green tea-based kombucha, with higher initial sugar concentration (94 g/L versus 63 g/L) revealed sweeter, thicker, and fruity aromatics [16]. Similarly, a significant relationship between sucrose and the overall liking for black tea kombucha has been observed under fermentation at 20 °C and 30 °C, respectively, which indicated that the highest mean liking scores could be obtained at 75 g/L sugar content [12]. These prior works have provided useful information on improving sensorial properties of tea-based kombucha by regulating the initial sugar concentration, while there is still a scientific gap in understanding its impacts on microbial community succession and metabolite changes [15,16].

Raw Pu-erh tea (RAPT) is a type of tea made from the sundried leaves of *Camellia sinensis* var. *assamica* and has been developed as substrate for kombucha production in our previous study [17]. Due to the important role of sugar content on kombucha fermentation, the aim of the present study is to evaluate the effect of initial sugar content on sensory properties of RAPT kombucha with the potential forming mechanism proposed. Thus, variation in sensory characteristics, volatile flavor profile, and microbial communities of RAPT kombucha were assessed under different initial sugar contents. Moreover, cooperative combination of macrogenomic and non-targeted metabolomic analysis was utilized to further elucidate the metabolite profile and pathways of fermented RAPT kombucha for optimizing sugar addition in kombucha production.

## 2. Materials and Methods

### 2.1. Materials and Chemicals

The RAPT materials were sourced from the Yunnan Pu-erh Tea Factory (Lincang, China). The kombucha inoculum was supplied by Yijianyuan Biomedicine Co., Ltd. (Jining, China), selected from a previous study and preserved at 4 °C until use [17]. Sucrose, used as the carbohydrate substrate, was obtained from the Shanghai First Food Mall (Shanghai, China). Standard *n*-alkanes (C_7_–C_30_) were purchased from Sigma-Aldrich (Shanghai, China). All other reagents were of analytical grade and supplied by Shanghai Meryl Biochemical Technology Co., Ltd. (Shanghai, China).

### 2.2. RAPT Kombucha Sample Preparation

Kombucha was produced from RAPT materials supplemented with sucrose following a previously described protocol with minor modifications [17]. In brief, RAPT materials (8 g/L) were infused in hot water (90 °C) for 15 min, after which the tea leaves were removed, and varying amounts of sucrose were added to prepare the sweetened tea extract. The resulting infusion was then inoculated with 5% (*v*/*v*) kombucha starter and incubated statically at 28 °C for 6 days. Four types of different kombucha samples were prepared to evaluate the substrate component especially for initial sugar content on sensory characteristics of RAPT kombucha. The RAPT kombucha sample 1 (S1) was prepared using kombucha starter fermented with 78 g/L sugar solution that free of RAPT materials. Sample 2 (S2) was prepared using kombucha starter fermented with 8 g/L tea infusion that free of sucrose. The RAPT kombucha sample 3 (S3) was prepared using kombucha starter fermented with sugared tea infusion (initial sucrose concentration of 39 g/L) used as substrate. Sample 4 (S4) was prepared using sugared tea infusion with initial sucrose concentration of 78 g/L, which was supposed to be the optimal initial sugar content used for RAPT kombucha fermentation based on preliminary sensory evaluation experiments (Tables S1 and S2). Sample preparation was conducted in triplicate for subsequent analysis.

### 2.3. Volatile Compound Analysis of RAPT Kombucha

The volatile compounds of RAPT kombucha were extracted by headspace solid-phase microextraction (HS–SPME) and analyzed by gas chromatography–mass spectrometry (GC–MS). Specifically, 6.5 g of kombucha was combined with 2.0 g NaCl in a 20 mL headspace vial, and 5 μL of 1,2-dichlorobenzene (100 ppm in acetone) served as the internal standard. The vial was placed on the autosampler (MPS robotic, Gerstel GmbH & Co. KG, Mülheim an der Ruhr, Germany) and equilibrated at 60 °C for 20 min. Volatiles were adsorbed with a DVB/CAR/PDMS (50/30 μm) SPME fiber (Supelco, Bellefonte, PA, USA) for 50 min and desorbed in the GC injector at 250 °C for 10 min [17]. GC–MS analysis was performed on an Agilent 8860A gas chromatograph coupled to a 5977B mass spectrometer (Agilent Technologies, Santa Clara, CA, USA), with separation on an HP-INNOWAX capillary column (60 m × 0.25 mm × 0.25 μm; Agilent Technologies, USA). High-purity helium (>99.99%) was used as the carrier gas. The chromatographic conditions were temperature programmed as follows: 40 °C for 3 min; ramp to 60 °C at 2 °C/min; then to 200 °C at 4 °C/min; and finally to 230 °C at 5 °C/min with a 15 min hold. The carrier gas flow was 1.0 mL/min with a 5:1 split ratio. The mass spectrometer was operated in full scan mode (30–450 *m*/*z*) at 70 eV with an ion source temperature of 230 °C [17].

### 2.4. Sensory Evaluation of RAPT Kombucha

The sensory evaluation of RAPT kombucha was performed with the quantitative descriptive analysis according to previous reported methods used in tea-based kombucha with slight modification [12,18]. The sensory evaluation experiments were conducted in the sensory laboratory and received approval from the Ethics Committee of the Shanghai Institute of Technology (Shanghai, China), with all participants provided and signed the informed consent. Prior to sensory evaluation, all participants were graduate students and laboratory staff members from the School of Perfume and Aroma Technology underwent calibration training with reference standards. The 10 panelists (6 females and 4 males, aged 23–35) were chosen who regularly participate in sensory studies of tea and fermented beverages. Firstly, participants were asked to taste each sample to familiarize themselves with its aroma attributes. Seven sensory descriptors (sour, floral, fruity, sweet, woody, fatty, and alcoholic) were then chosen and defined during a panel discussion for use in quantitative descriptive analysis. The reference standards applied were acetic acid (sour), phenylethyl alcohol (floral), ethyl acetate (fruity), vanillin (sweet), cedrol (woody), nonanal (fatty), and ethanol (alcoholic). Aroma intensity for each descriptor was rated on a 9-point scale (0–1, weaker; 2–3, weak; 4–5, middle; 6–7, strong; 8–9, stronger). For sensory testing, 50 mL of each kombucha sample was poured into a 150 mL odor-free plastic sensory cup at room temperature and labeled with a random three-digit code. Panelists were then instructed to evaluate the samples and record the intensity ratings for each descriptor. A 5 min break was provided between evaluations for sensory recovery, and every kombucha sample was assessed in triplicate. After tasting all tested samples, the overall liking of RAPT kombucha samples was further evaluated through score ranking according to the previous reported method [12].

### 2.5. Microbial Analysis of RAPT Kombucha by Plate Count Methodology and Illumina High-Throughput Sequencing

The microbial plate counting was performed according to the methodology reported previously [10]. The total DNA of SCOBY was extracted and isolated with slight modifications made based on the previously reported method [19]. Firstly, 50 mL fermented kombucha sample was collected and centrifuged at 4 °C and 5000 rpm for 15 min to obtain the microbial pellets. The pellets were washed with deionized water and resuspended in 1 mL of lysis buffer solution, and the total DNA was extracted using FastDNA^®^ SPIN Kit (MP Biomedicals, Santa Ana, CA, USA) followed according to the manufacturer’s protocol. The extracted DNA was analyzed by 1% (*w*/*w*) agarose gel electrophoresis, and its concentration and purity were determined using the OD260/OD280 ratio measured with a NanoDrop 2000 spectrophotometer (Thermo Scientific, Waltham, MA, USA). The total DNA fragments of the samples were split into approximately 400 bp long segments using a M220 focused ultrasound instrument (Covaris Inc., Woburn, MA, USA). The primers 338F (5′-ACT CCT ACG GGA GGC AGC AG-3′) and 806R (5′-ACT CCT ACG GGA GGC AGC AG-3′) were used to amplify the V3–V4 variable region of bacterial 16S rRNA gene in a thermocycler (GeneAmp 9700, ABI, Foster City, CA, USA) as described previously [20]. The V1 region with ITS5 (GGA AGT AAA AGT CGT AAC AAG G) and ITS2 (GCT GCG TTC TTC ATC GAT GC) were used as primers to amplify the fungal ITS genes [21]. The PCR products were purified with the AxyPrep DNA Gel Extraction Kit (Axygen Biosciences, Union City, CA, USA). The high-throughput sequencing was carried out on the Illumina MiSeq platform (San Diego, CA, USA) by Majorbio Bio-Pharm Technology Co., Ltd. (Shanghai, China). Sequencing libraries were prepared using the NEXTFLEX™ Rapid DNA-Seq Kit (Bioo, Austin, TX, USA) together with NovaSeq Reagent Kits (Illumina, San Diego, CA, USA), following the manufacturer’s protocols. Raw reads generated on the NovaSeq 6000 platform (Illumina, USA) were processed into Fastp format, and quality filtering was applied to eliminate low-quality reads (<50 bp), yielding clean data for accurate downstream analyses. Bioinformatic processing of 16S rRNA and ITS sequences was performed with UCHIME as previously described [22].

### 2.6. Macrogenomic and Non-Targeted Metabolomic Analysis of Kombucha Fermentation Under Optimal Initial Sugar Content

#### 2.6.1. Microbial Analysis of RAPT Kombucha by Illumina High-Throughput Sequencing

The total DNA was extracted as described in 2.5 and sequences were checked using BWA (version 0.7.17) by comparison of sequencing results with the parasitifer DNA sequences to remove contaminating reads of high similarity. The quality-checked reads were then assembled into contigs using MEGAHIT (https://github.com/voutcn/megahit, version 1.0, accessed on 24 January 2024), and the prediction was performed based on the contigs obtained from the spliced results using Prodigal (https://github.com/hyattpd/Prodigal, version 2.6.3, accessed on 24 January 2024). The cluster analysis (95% identity and 90% coverage) was conducted using CD-HIT version 4.8.1, and the longest gene from each cluster was selected as the representative sequence to generate non-redundant gene sets. The BLAST (http://blast.ncbi.nlm.nih.gov/Blast.cgi, version 2.2.28, accessed on 29 January 2024) was applied to align these non-redundant sets against the eggNOG database (eggNOG, https://eggnogdb.embl.de/#/app/home, accessed on 29 January 2024), the non-redundant protein sequence database (NR), the Kyoto Encyclopedia of Genes and Genomes database (KEGG, https://www.kegg.jp/kegg/, accessed on 29 January 2024) and the carbohydrate active enzymes database (CAZy, https://www.cazy.org/, accessed on 29 January 2024) for abundance estimation and functional annotation [23]. Microbial diversity and metagenomic datasets were further processed using the Majorbio Cloud platform (www.majorbio.com, accessed on 2 February 2024), following previously described methods [17].

#### 2.6.2. Non-Targeted Metabolomic Analysis by UPLC-Q-TOF-MS

With initial sugar content of 78 g/L, the RAPT kombucha sample was collected on day 0 (K0) and day 6 (K6), respectively, and centrifuged to obtain supernatant solution. The supernatant solution was firstly freeze-dried and 50 mg of which was redissolved in 0.4 mL of 50% methanol containing internal standards. After stand at 4 ℃ for 30 min, the redissolved solution was centrifuged at 4 °C for 15 min to remove precipitate for analysis, with redissolved solution taken from each sample mixed (20 μL × 6) and used as quality control (QC) samples to assess the reliability of the analyzed results. For metabolomic analysis, the instrument was consisted of a UHPLC-Q Exactive system (Thermo Fisher Scientific, Waltham, MA, USA) coupled to a time-of-flight mass spectrometer (TOF-MS) (Thermo Fisher Scientific, USA). The sample was separated by an ACQUITY UPLC BEH C18 column (100 mm × 2.1 mm i.d., 1.7 µm) (Waters, Milford, CT, USA). The mobile phase was consisted of solvent A water (2% acetonitrile and 0.1% formic acid) and solvent B acetonitrile (0.1% formic acid), with the elution gradient procedure programmed as follows: 2% B (98%A) at 0–0.5 min, 2–35% B at 0.5–7.5 min, 35–95% B at 7–13 min, and 2% B at 13–14.5 min. The analysis was performed with injection volume of 3 µL, flow rate of 0.4 mL/min, and column temperature of 40 °C. The ion source was electrospray ionization (ESI), and the mass spectrometry signals were collected in positive and negative ion scanning modes, respectively, with the spray voltage in the positive mode of 3500 V and in the negative mode of −3000 V. The samples were analyzed in the scanning range of 70–1050 (*m*/*z*) and the collision energy range of 10–40 eV. Three QC samples were run to equilibrate the column before the injection of samples, ion peaks with RSD > 0.3 in the QC group were deleted, and each sample was analyzed five times [24].

#### 2.6.3. Metabolomics Data Analysis and Function Annotation

The raw data of UPLC-Q-TOF-MS were pre-processed using Progenesis QI, version 3.1 (Waters Corporation, Milford, CT, USA) software. The internal standard peaks and false positive peaks in the raw data were removed to obtain non-redundant data. Metabolites were also identified by searching the Majorbio plant-specific metabolite database (MJDBPM) (https://cloud.majorbio.com/, accessed on 2 February 2024). Metabolites were screened to exclude quality control sample variables with relative standard deviation (RSD) greater than 30%, the features of ion peaks with >50% missing values were excluded, and the minimum values of each group were calculated to substitute the missing values [25]. Finally, the positive and negative ion data were normalized and merged to create a data matrix table for further analysis.

Perform variance analysis was performed using the R package “Ropls” (Version 1.6.2) for the principal component analysis (PCA) and orthogonal least partial squares discriminant analysis (OPLS-DA). The variable importance in projection (VIP) coefficients obtained by the OPLS-DA were regarded as a variable that contributes to data description, reflecting the relative importance of each independent variable. The *p*-value was generated by a two-tailed Student’s *t*-test, and potential metabolites were selected by the standard of *p*-value < 0.05 (*t*-test) and VIP value > 1 from the OPLS-DA model [25,26]. Differential metabolites between the two groups were annotated to their biochemical pathways using metabolic enrichment and pathway analysis based on the KEGG database (http://www.genome.jp/kegg/, accessed on 2 February 2024) [26]. Enrichment analysis was conducted with the Python package “Scipy.stats” (https://docs.scipy.org/doc/scipy/, accessed on 2 February 2024) to identify the biological pathways most closely associated with the experimental treatments.

### 2.7. Statistical Analysis

All measurements including multi-omics, GC-MS, and sensory analysis were conducted in triplicate, with results expressed as the mean ± standard deviation. Statistical analyses were conducted with IBM SPSS Statistics v20 (IBM SPSS Inc., Chicago, IL, USA) using ANOVA, and Duncan’s multiple range test was applied to assess differences among samples. Significance was determined at *p* < 0.05, and highly significant differences were noted at *p* < 0.01. Origin 2023 (MicroCal, Northampton, MA, USA) was used for PCA, as well as for generating heat maps and radar plots.

## 3. Results

### 3.1. Volatile Profiles of Different RAPT Kombucha Samples

**Figure 1:**
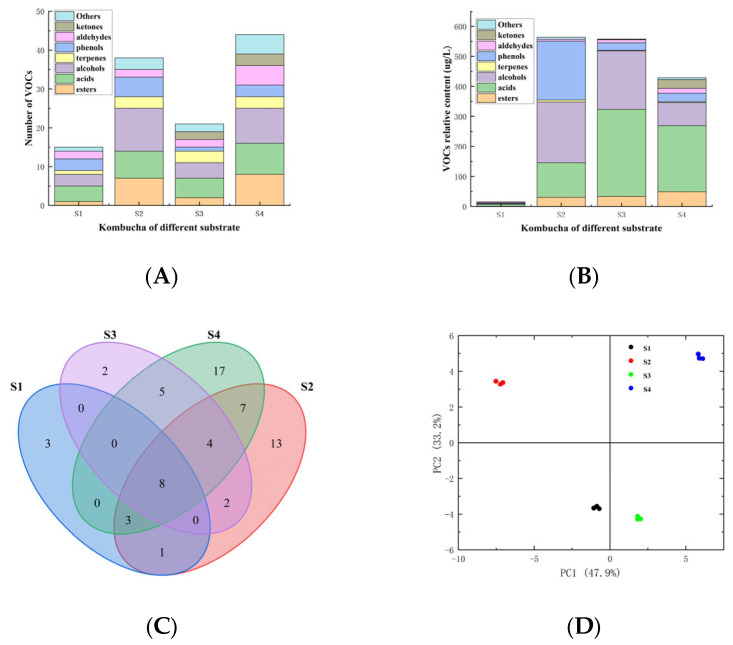
Comparison of volatile profiles of kombucha fermented with different concentration of sugar. (**A**) Number of VOCs; (**B**) VOCs relative content; (**C**) Veen diagram analysis; (**D**) PCA score of the four samples stemmed from GC-MS. S1: 78 g/L sugar solution that free of tea materials fermented with SCOBY for 6 days, S2: 8 g/L tea materials that free of sugar fermented with SCOBY for 6 days, S3: 8 g/L tea and 39 g/L sugar concentration fermented with SCOBY for 6 days, S4: 8 g/L tea and 78 g/L sugar concentration fermented with SCOBY for 6 days.

After 6 days of fermentation, the volatile organic compound (VOC) contents of four different types of RAPT kombucha samples were determined by HS-SPME-GC-MS. A total of 65 VOCs were identified in all the four tested samples (Table S1). These volatiles including 10 esters, 4 terpenes, 9 acids, 16 alcohols, 4 ketones, 6 phenols, 7 aldehydes, and 8 other compounds, with 15 volatile compounds identified in S1, 38 compounds in S2, 21 compounds in S3, and 44 compounds in S4 (Figure 1A). Moreover, the differentiation in VOCs content of RAPT kombucha was also observed among the four samples fermented with different initial sugar (tea) concentration. As shown in Figure 1B, the total VOCs content detected in S2 was the highest (563.91 µg/L), followed by S3 (557.81 µg/L), S4 (428.88 µg/L), and S1 (15.46 µg/L), respectively. Compared to other three RAPT kombucha samples, the VOCs content of S1 that free of tea materials in substrate was the lowest, indicating tea components are of great importance for the formation of aroma compounds. When tea infusion was used as substrate, the VOCs content of sample S2 (substrate free of sugar) was comparable to S3 (initial sugar content of 39 g/L) and much higher than that of S4 (initial sugar content of 78 g/L). However, the volatile compounds varied considerably among each sample as exhibited in Veen diagram analysis and principal component analysis (Figure 1C,D). For example, 8 volatiles were detected in all the four samples among the 65 identified compounds based on the result of Venn diagram analysis (Figure 1C). Meanwhile, the PCA result (PC1 accounted for 47.9%and PC2 accounted for 33.2%, respectively) revealed that the four samples were situated in disparate regions, indicating that great distinctions in volatiles could be observed among these samples (Figure 1D). Additionally, more phenols were contained in S2 while less detected in S3 and S4, and S3 revealed significant (*p* < 0.05) higher acids and alcohol content than S4 (Table S3). During the fermentation process, alcohol could be formed from sugar by yeasts and further responsible for creating organic acids by acetic acid bacteria (AAB) metabolism, which are both important metabolites for the sensory properties of kombucha [1,18]. The result here suggested that initial sugar concentration played vital role in biosynthesis of organic acids and alcohol in microorganisms, and therefore could be important parameters used for regulating the sensory characteristics of RAPT kombucha.

The odor activity value (OAV), defined as the ratio of a volatile compound’s concentration to its odor detection threshold in water, was used to evaluate its contribution to the overall aroma of RAPT kombucha. According to previous reports, compounds with OAVs ≥ 1 are generally regarded as key contributors to the aroma profile [27]. In all kombucha samples tested, eight aroma-active compounds with OAV ≥ 1 were identified (Table 1). The fermented sample S1 had no aroma compound with OAV ≥ 1 when sugar solution that free of tea leaves used as substrate. Differ from S1, S2 that use only tea infusion as substrate (free of sugar content) had 5 aroma active compounds, including linalool (OAV = 371.91), eucalyptol (OAV = 4.06), geraniol (OAV = 3.51), butanoic acid (OAV = 127.62), and 4-ethylphenol (OAV = 9.64). Among these compounds, linalool and geraniol were recognized as aroma-active compounds of RAPT infusion previously [27], suggesting that other 3 compounds (eucalyptol, butanoic acid, and 4-ethylphenol) might be newly formed during the fermentation process. When sugar and tea materials were both supplemented as substrate for kombucha preparation, the sample S3 with low initial sugar concentration (3.9 g/L) exhibited 3 aroma-active compounds, including linalool (OAV = 80.82), geraniol (OAV = 5.98), and ethyl acetate (OAV = 4.06). The OAVs of these three compounds were in general agreement with previous reported values during the RAPT kombucha fermentation process [1]. Additionally, the sample S4 with high initial sugar concentration (7.8 g/L) had 5 aroma-active compounds including linalool (OAV = 41.95), ethyl acetate (OAV = 3.15), *β*-damascenone (OAV = 270.42), 4-ethylphenol (OAV = 1.32), and 2,4-decadienal (OAV = 30.00) (Table 1). Compared to sample S2 and S3, S4 possessed the highest initial sugar concentration and exhibited three differential aroma-active compounds including ethyl acetate, β-damascenone, and 2,4-decadienal (Table 1), which might contribute to the fruity, floral and fatty aroma of RAPT kombucha, respectively [17]. Several of the identified aroma-active compounds can be linked to specific microbial pathways. For instance, ethyl acetate is formed by yeasts through the esterification of ethanol and acetyl-CoA, while *β*-damascenone originates from the degradation of carotenoids facilitated by microbial activity [1,7,27]. In addition, 4-ethylphenol is commonly associated with *Brettanomyces*, which metabolizes hydroxycinnamic acids, and 2,4-decadienal may derive from lipid oxidation [28,29]. Different content of these compounds detected in S3 and S4 demonstrated that initial sugar concentration could shape the accumulation of aroma-active volatiles in RAPT kombucha, which was attribute to the variation in microbial growth and metabolism caused by initial sugar concentrations. Furthermore, some volatile compounds with OAV < 1 may also influence the aroma perception of RAPT kombucha through synergistic or additive effects. The interactions among volatiles with OAV < 1 should be further investigated to better understanding the aroma complexity of RAPT kombucha.

### 3.2. Sensory Characteristics of Different RAPT Kombucha Samples

**Figure 2:**
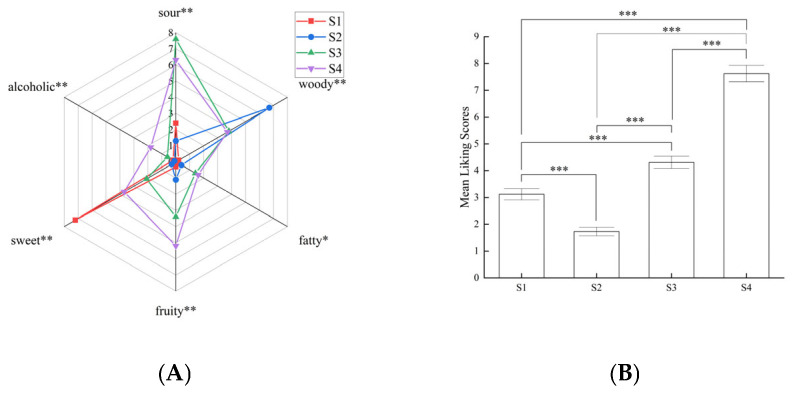
Evaluation of sensory characteristics of four kombucha samples. (**A**) the aroma sensory descriptors of the four RAPT kombucha samples; (**B**) mean liking scores of four kombucha samples. S1: 78 g/L sugar solution that free of tea materials fermented with SCOBY for 6 days, S2: 8 g/L tea materials that free of sugar fermented with SCOBY for 6 days, S3: 8 g/L tea and 39 g/L sugar concentration fermented with SCOBY for 6 days, S4: 8 g/L tea and 78 g/L sugar concentration fermented with SCOBY for 6 days. * *p* < 0.05, ** *p* < 0.01, *** *p* < 0.001.

The sensory evaluation of each sample was performed after 6 days of SCOBY fermentation, with sensory descriptors (sweet, sour, woody, fruity, alcoholic, and fatty) were selected based on the results of quantitative descriptive analysis reported previously [30]. As shown in Figure 2A, the aroma sensory descriptors of the four RAPT kombucha samples were significantly different (*p* < 0.05 and *p* < 0.01). Specifically, the substrate of S1 was free of tea materials and the fermented sample only revealed strong sweet (7.2) and week sour (2.4) sensory intensities, and S2 that fermented with tea infusion (initial sugar concentration = 0) mainly revealed strong woody intensity (6.7) while lack of other sensory characteristics (Figure 2A). The substrate of S1 (S2) was lack of nitrogen (or carbon) source, which was unable to provide sufficient nutrients for the development of SCOBY and therefore would result in formation of less flavor substances [31]. For S3 and S4, carbon and nitrogen sources were both sufficient for the kombucha fermentation, and more sensory characteristics (alcoholic, fruity, fatty) were exhibited as compared to nutrition deficiency sample S1 and S2 (Figure 2A). The sensory evaluation revealed that more sour intensity was observed in S3 as compared with S4, which was reasonably in agreement with the results of GC-MS that more organic acids could be formed under low initial sugar concentration (Figure 2A and Table S3). However, S4 exhibited higher score (*p* < 0.01) on sweet, alcoholic, fruity, and fatty intensities as compare to S3 (Figure 2A). On case of overall liking of RAPT kombucha samples, both S1 and S2 exhibited low scores (3.1 and 1.7, respectively), which might due to the lack of nitrogen/carbon source contained in substrate (Figure 2B). Additionally, S4 revealed higher score (7.6) as compared with S3 (4.3), which might attribute to the relatively decreased woody flavor and significantly improved sensory intensities of alcoholic, fruity, and fatty (Figure 2A,B). The result indicated that the increased initial sugar content could contribute to the improvement of sensory properties.

### 3.3. Illumina High-Throughput Sequencing Analysis of S3 and S4

**Figure 3:**
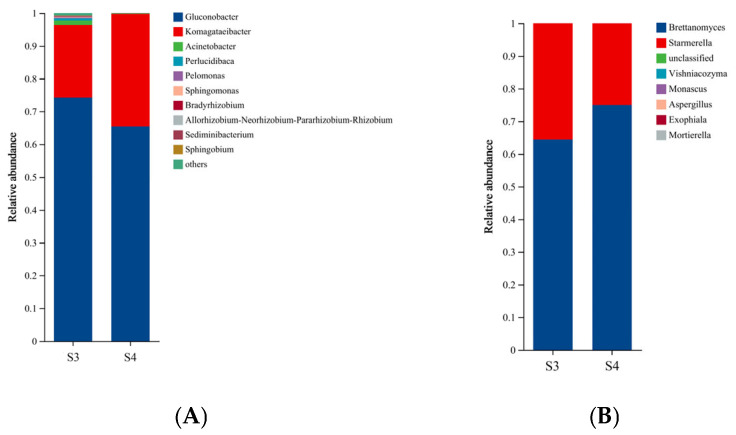
Relative abundance of the microbial communities in S3 and S4 samples. (**A**) Bacterial communities; (**B**) Fungal communities. S3: 8 g/L tea and 39 g/L sugar concentration fermented with SCOBY for 6 days, S4: 8 g/L tea and 78 g/L sugar concentration fermented with SCOBY for 6 days.

The final quality of kombucha largely depends on the microbial composition and dynamic succession during the fermentation process [19,21]. Before conducting sequencing work, the plate counting method was used to count yeast, AAB, and mold in four kombucha samples according to the method reported previously [10]. As shown in Table S4, no mold was detected in any of the samples, and S1 and S2 maintained low total counts levels. The total counts of yeast and acetobacter in S4 was higher than that in S3. The Illumina high-throughput sequencing analysis was further conducted on kombucha samples, as it was unable to identify the microbial genus or species. As shown in Figure 3A, *Gluconobacter* and *Komagataeibacter* were the most dominant bacterial genera in both samples, with a small proportion of other bacterial genera such as *Acinetobacter* and *Perlucidibaca* existed. The result here was consistent with previous foundations that *Gluconobacter* and *Komagataeibacter* were common dominant bacterial genera observed in SCOBY [21,28]. Compared with S3, the increased initial sugar concentration in S4 could upregulate the proportion of *Komagataeibacter* and downregulate the proportion of *Gluconobacter* as well as other minor bacterial genera (Figure 3A). Meanwhile, the Illumina high-throughput sequencing analysis revealed that *Brettanomyces* and *Starmerella* were the most dominant fungal genera detected in both S3 and S4, with the proportion of *Brettanomyces* genus higher contained in S4 and *Starmerella* higher contained in S3 (Figure 3B). The high proportions of *Brettanomyces* and *Starmerella* genera in fermented kombucha have also been reported in other SCOBY originated in China [17,21], while *Candida*, *Hanseniaspora*, and *Zygosaccharomyces* revealed to be dominant fungus of SCOBY derived from other origin areas [32]. Based on the aforementioned information of high initial sugar concentration on volatile profiles and sensory characteristics (Figure 1 and Figure 2), bacteria *Komagataeibacter* and yeast *Brettanomyces* might serve important roles on regulating aroma-active compound metabolism and enhancing the sensory quality of RAPT kombucha. These results indicated that microbial succession could be significantly influenced by the initial sugar concentration, which would result in the formation of different metabolites and lead to various sensory characteristics.

### 3.4. Metagenomic and Metabolomic Analysis Under Optimal Initial Sugar Concentration

The RAPT kombucha S4 with initial sucrose content of 78 g/L exhibited the highest liking score among the four tested samples, which was supposed to be the optimal initial sugar concentration for the fermentation based on sensory guided optimization process (Figure 2 and Table S1). Herein, metagenomic and metabolomic analysis was performed for better understanding the influence of optimal initial sugar concentration on the desired sensory profile of RAPT kombucha.

#### 3.4.1. Metagenomic Analysis

**Figure 4:**
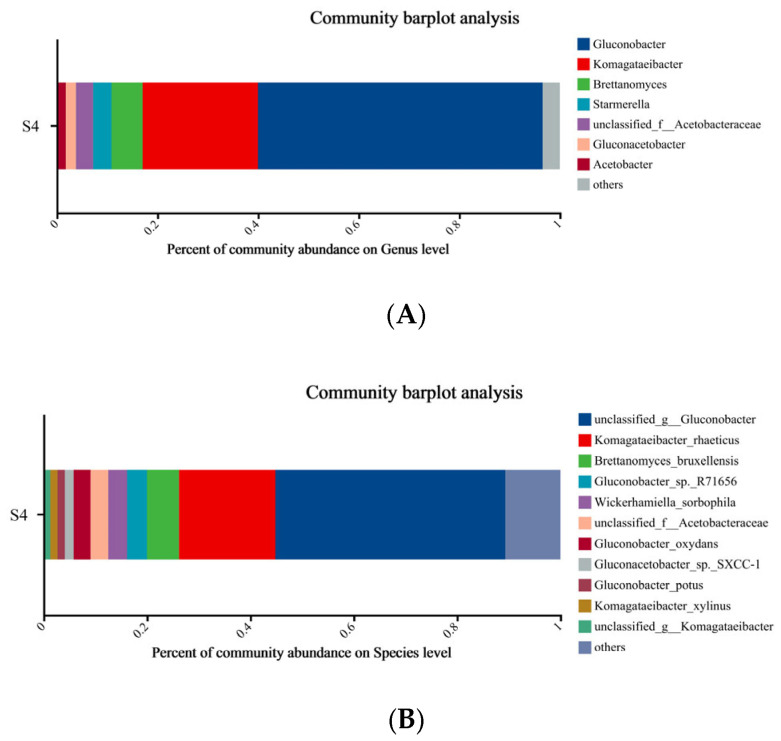
Composition of dominant microbial genera/species (RA > 1%) of S4 kombucha sample. (**A**) genera level; (**B**) species level. S4: 8 g/L tea and 78 g/L sugar concentration fermented with SCOBY for 6 days.

At the genus level, the dominant genera of AAB that were identified were *Gluconobacter*, *Komagataeibacter*, *Gluconacetobacter*, and *unclassified f Acetobacteraceae*, while *Brettanomyces* and *Starmerella* were the dominant genera of yeasts observed in SCOBY (Figure 4A). As shown in Figure 4A, the most abundant genus was *Gluconacetobacter*, whose sequences accounted for 56.6% of the sample, followed by *Komagataeibacter* (22.9%). Meanwhile, *Brettanomyces* and *Starmerella* were the two most abundant yeast genera with an average relative abundance of 6.1% and 3.6%, respectively (Figure 4A). At the species level, there were 11 species with average relative abundance higher than 1% (Figure 4B). The dominant species contained in the S4 were *unclassified g Gluconobacter*, *Komagataeibacter rhaeticus*, *Brettanomyces bruxellensis*, and *Gluconobacter* sp. *R71656*, which was similar to the previous observation in the SCOBY obtained from France [33]. After the later period of fermentation, SCOBY formed metabolites such as alcohols that could suppress the growth of yeasts, while AAB such as *Komagataeibacter* might utilize these substances for acid production [34]. Therefore, the relative abundance of AAB was much higher than yeast species after 6 days of fermentation (Figure 4).

**Figure 5:**
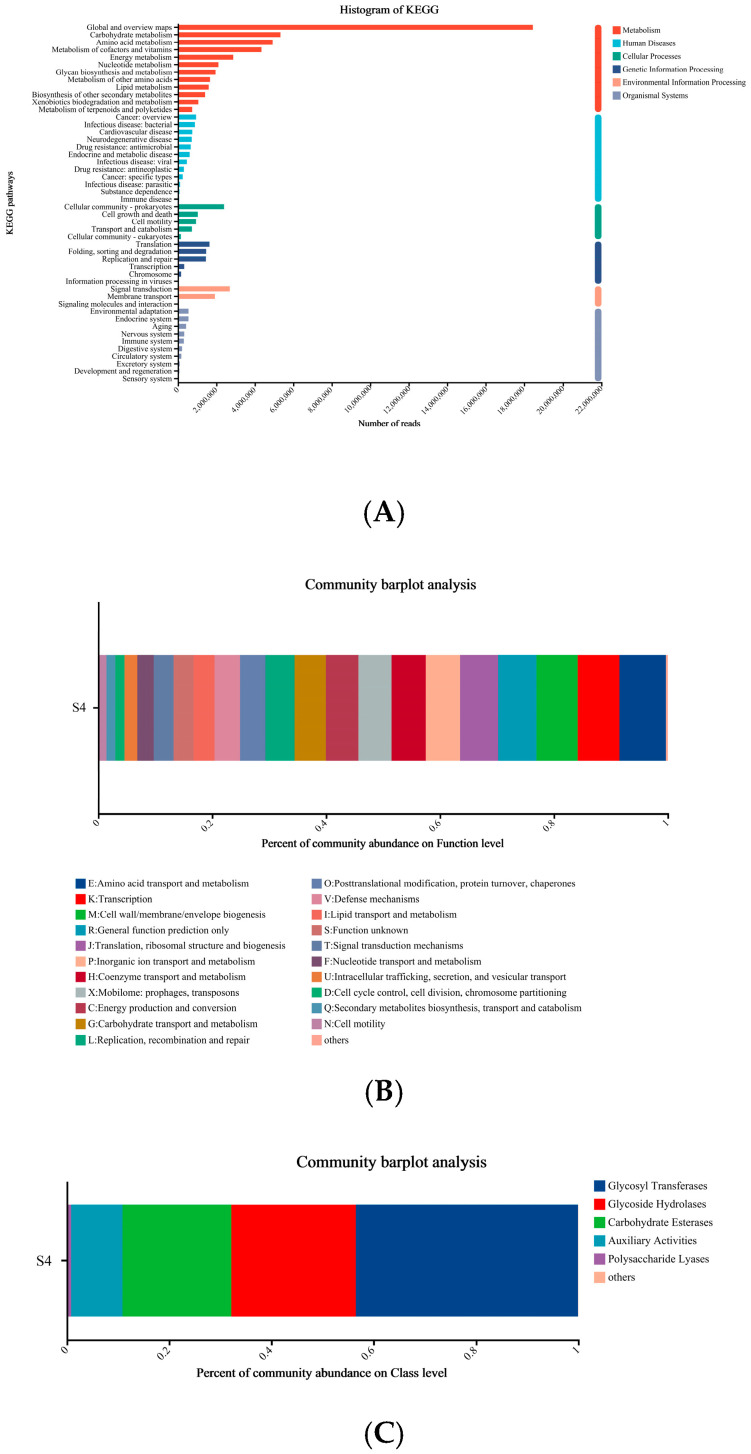
Gene annotation results of S4 kombucha sample. (**A**) KEGG databases; (**B**) eggCOG database; (**C**) Cazy database. S4: 8 g/L tea and 78 g/L sugar concentration fermented with SCOBY for 6 days.

The KEGG, CAZy and eggNOG databases (e-value ≤ 1 × 10^−5^) were used to functionally annotate the S4 samples. As shown in Figure 5A, the vertical axis indicates the KEGG Pathway Level 2 functional categories, while the horizontal axis shows their corresponding abundance values. The KEGG Pathway Level 2 is colored in a bar chart according to the KEGG Pathway Level 1. In the KEGG Pathway (Level 1), all the genes were categorized into 6 classes (Figure 5A) with the highest proportion of genes related to metabolism, which reached 49.72% of the total number of genes in the KEGG Pathway. Within the Level 2 pathway, the most prevalent pathways in SCOBY were global and overview maps, followed by carbohydrate metabolism, amino acid metabolism, and metabolism of cofactors. The carbohydrate metabolism pathway in SCOBY contributed an average of 7.62% to the total microbial gene abundance (Figure 5A). It is well known that sugar degradation is associated with carbohydrate metabolism in biosynthesis of ethanol and acetic acid, which are important flavor substances during the kombucha fermentation process [33]. The eggNOG analysis revealed that the genes in the S4 were grouped into 22 categories (Figure 5B), with the first 6 categories being E (Amino acid transport and metabolism), K (Transcription), M (Cell wall/membrane/envelope biogenesis), R (General function prediction only), J (Translation, ribosomal structure and biabolism), and P (Inorganic ion transport and metabolism). Of these, E was the major metabolic gene, which was consistent with the annotation results of the KEGG data. This indicated that amino acid metabolism might be greatly impacted by the SCOBY during the fermentation. In addition, the SCOBY genes were then compared with the CAZy database to predict potential genes involved in carbohydrate metabolism. As shown in Figure 5C, all genes were categorized into 6 groups, in order of Glycosyl Transferases (GT), Glycoside Hydrolases (GH), Carbohydrate Esterases (CE), Auxiliary Activities (AA), Polysaccharide Lyases (PL) and others. GTs catalyze the attachment of intracellular sugars to different receptor molecules, resulting in the synthesis of oligosaccharides and polysaccharides, which play a key role in the sugar metabolism [35]. GHs are important enzymes for the hydrolysis of sugars, which converts sucrose to monosaccharides, providing AAB with a source of carbon that can be utilized. CEs are another large group of carbohydrate-active enzymes that catalyze the removal of ester substituents of carbohydrates and polysaccharide chains. AA plays an important role for the degradation of plant cellulose, and PL is a degrading enzyme of polysaccharides in its major domain of action [36]. These potential genes confirm the critical importance of the KEGG metabolic pathway in sugar metabolism, and these enzymes contained in the microbiota might also be associated with the formation of cellulose membranes in kombucha [17].

#### 3.4.2. Non-Targeted Metabolomic Analysis

**Figure 6:**
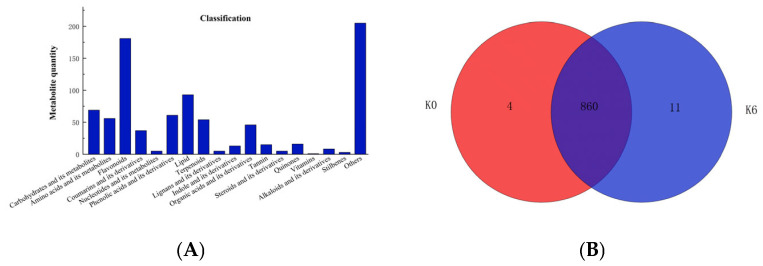
Metabolite annotation results of unfermented and fermented S4. (**A**) Cylindrical diagram of metabolite number classification; (**B**) Wayne diagram of the number of metabolites in different samples. K0: unfermented S4; K6: S4 fermented for 6 days.

In order to elucidate non-volatile changes under optimal sugar concentration and its potential correlation with microorganisms, non-targeted metabolomic analysis on S4 before and after fermentation (K0 and K6) was carried out by using the UPLC-Q-TOF-MS. As shown in Figure 6A, a total of 873 metabolites (pos 476, neg 397) were obtained after processing using Progenesis QI (Waters Corporation, Milford, CT, USA) software. These metabolites were annotated using the KEGG database and the HMDB database, yielding a total of 181 flavonoids, 69 carbohydrates and their derivatives, 56 amino acids and their derivatives, 37 coumarins and their derivatives, 5 nucleotides and their derivatives, 61 phenolic acids and their derivatives, 93 lipids, 54 terpenoids, 5 lignans and their derivatives, 13 indoles and their derivatives, 46 organic acids and their derivatives, 15 tannins, 5 steroids and their derivatives, 16 quinones, 1 vitamin and 8 quinones, 8 alkaloids and their derivatives, 3 astragalus and 205 others (Figure 6A). In terms of their proportions, flavonoids, others, esters, carbohydrates and their derivatives, phenolic acids and their derivatives, and amino acids and their derivatives were the five dominant metabolites detected (Figure 6A). The Venn analysis revealed that there were 864 metabolites detected in K0 and 871 metabolites detected in K6, respectively, with 860 metabolites observed in both groups (Figure 6B). Four compounds, including gingerglycolipid A, (2′E,4′Z,7′Z,8E)-vanillic acid, forcalcene alkynediol, and 19α-19-hydroxy-3,11-dioxo-12 ursen-28-oic acid were endemic to K0, and eleven compounds were endemic to K6 (Figure 6).

#### 3.4.3. Multivariate Statistical Analysis

Multivariate statistical analysis was performed using unsupervised analysis (PCA) and supervised analysis (OPLS-DA). For PCA, PC1 and PC2 explained 47.9% and 7.3% of the total variance, respectively (Figure S1A). Meanwhile, the quality control (QC) samples were centrally distributed and reproducible, indicating that the system was stable. The results showed that all samples were within the confidence ellipse, indicating that this data warrants further investigation. Therefore, a supervised method (OPLS-DA) was applied to better differentiate fermented from unfermented samples (Figure S1B). The permutation test (Figure S1C) showed Q^2^ (0.975) and R^2^ (0.993) Y values both approaching 1 without signs of overfitting, confirming that the OPLS-DA model was robust and exhibited a strong predictive performance [26].

**Figure 7:**
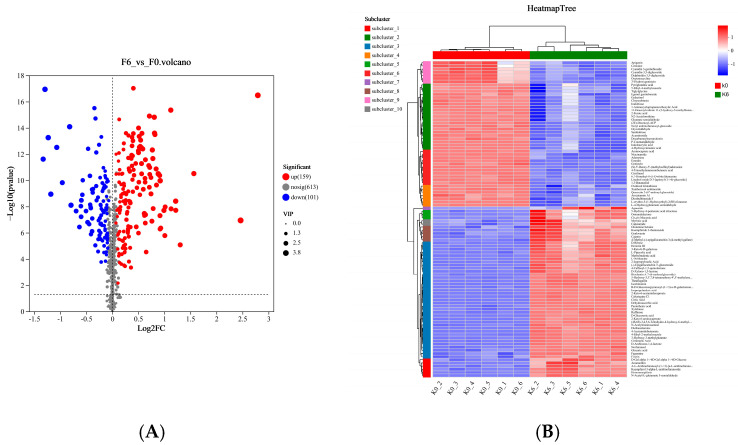
Metabolite annotation results of unfermented and fermented S4. (**A**) Cylindrical diagram of metabolite number classification; (**B**) Heatmap of metabolites of S4 before and after SCOBY fermentation. K0: unfermented S4; K6: S4 fermented for 6 days.

In the OPLS-DA model, the variable importance in projection (VIP) indicates the significance of each variable within the model and serves as a key parameter for evaluating the contribution of individual metabolites to the model’s classification performance [26]. To determine the differential metabolites between fermented and unfermented S4, metabolites with VIP ≥ 1 and *p* ≤ 0.05 in the OPLS-DA model were visualized in the form of volcano diagrams (Figure 7A and Table S5). A total of 260 different metabolites were obtained with VIP ≥ 1 (*p* ≤ 0.05), including 159 up-expressed metabolites and 101 down-expressed metabolites (Figure 7A and Table S5). The screened metabolic fractions were then compared with the KEGG compound database and HMDB database, and the fractions with definite names could be obtained. There were 145 differential metabolites in the positive ion mode, of which 82 were significantly upregulated and 63 were significantly downregulated (Figure 7A and Table S5). Additionally, there were 115 differential metabolites observed in the negative ion mode, of which 77 were significantly upregulated and 38 were significantly downregulated (Table S5). These differential metabolites were mainly flavonoids (isoflavone C, rhizophorin), lipids, vitamins (pantothenic acid, niacinamide), organic acids (*D*-glucuronic acid, caproic acid, butyric acid, citric acid, etc.), amino acids (*L*-tryptophan, ketoleucine, pyroglutamic acid, *L*-aspartic acid, etc.), and so on. The clustering heat map was used to more intuitively observe the relative content changes in the metabolites before and after fermentation. The differential metabolites with the expression in the top 50 of the metabolite set were clustered and analyzed (Figure 7B). As shown in Figure 7B, 12 samples (6 K0 and 6 K6, respectively) could be divided into two main branches, with unfermented samples (K0) and the fermented samples (K6) separated according to the clustering tree. This indicated that the metabolites between the K0 and K6 groups were very different and the two groups of samples could be well differentiated. In addition, all replicates of each group of samples were clustered into one branch, suggesting good uniformity and a high reliability of replicates of the same group. Furthermore, metabolites were clustered into 10 major branches based on the left clustering tree, indicating that the trend of expression changes in these metabolites was consistent among different sample groups (Figure 7B).

It can be seen from Figure 7B that the differential metabolites between K0 and K6 groups were mainly amino acids, carbohydrates, organic acids, flavonoids, lipids and some other classes of compounds. Among them, free amino acids are not only the main nitrogen source for microbial growth and metabolism, but also react directly with sugars to produce aroma components [21]. Compare with K0 group, the content of amino acids in K6 decreased after 6 days of fermentation by SCOBY, which suggested that microorganisms might use amino acids for growth/reproduction and the production of newly formed metabolites including flavor compounds. A similar phenomenon was also observed in the content of carbohydrates (Figure 7B). Additionally, organic acids are considered to be important contributors to the sensory property of kombucha, which could be produced from sugar by yeast and AAB through the EMP pathway, TCA cycle and some other metabolic pathways [29]. Besides the sensory contribution, some organic acids have health-promoting benefits, such as glucuronic acid, which is believed to have an important effect on the antioxidant and detoxification properties of kombucha [21]. However, excessive acetic acid content in kombucha would exhibit a pungent sour flavor and reduce sensory pleasure; therefore, adding moderate amounts of malic and citric acids can attenuate the irritation of acetic acid and make the taste of kombucha beverage softer, mellower and richer [37]. For the fermented S4, the relative low sour intensity as compared with S3 and the highest liking score suggested that the formation of organic acids could be greatly impacted by the initial sugar concentration (Figure 2B and Figure 7B).

### 3.5. Analysis of Differential Metabolites Under Optimal Sugar Concentration

**Figure 8:**
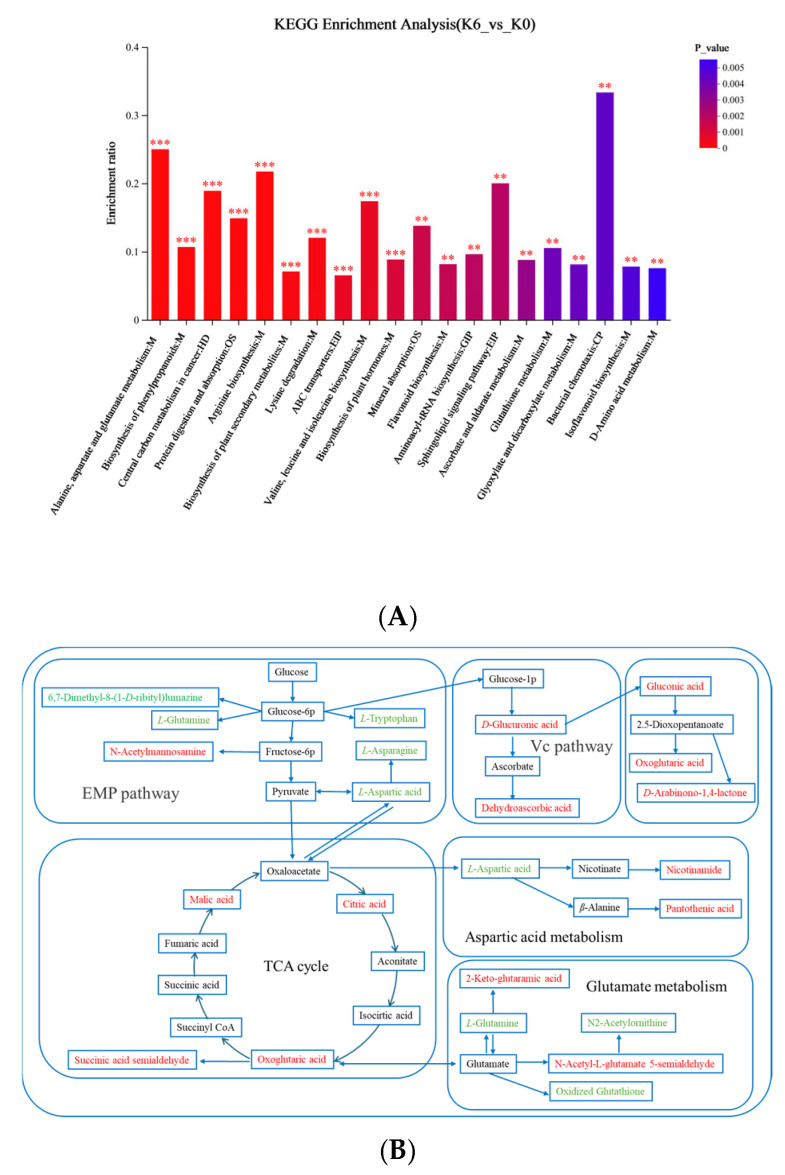
Differential metabolic pathway analysis of unfermented and fermented S4. (**A**) KEGG pathway enrichment analysis diagram of differential metabolic components; (**B**) Schematic diagram of key metabolic pathways of differential metabolites in kombucha. K0: unfermented S4; K6: S4 fermented for 6 days. ** *p* < 0.01, *** *p* < 0.001.

The KEGG enrichment pathway analysis was employed to better understand the key metabolites and metabolic pathways of S4 before and after SCOBY fermentation, with the metabolic pathways of differential metabolites analyzed using Scipy (Python) Version 1.0. As shown in Figure 8A, the horizontal coordinate represents the pathway name, the vertical coordinate indicates the ratio of the number of metabolites enriched into the pathway to the number of metabolites annotated to the pathway (i.e., the enrichment ratio), and the color gradient of the columns indicates the significance of the enrichment. The results showed that a total of 20 pathways (*p* < 0.05) were obtained, with the highest enrichment ratio revealed to be the bacterial chemotaxis (Figure 8A). During kombucha fermentation, pathways including alanine, aspartate, and glutamate metabolism, arginine biosynthesis, and the citric acid cycle are crucial to produce flavor and bioactive compounds (Figure 8A). Nevertheless, the generation of these compounds is complex due to the numerous reactions involved. To further investigate the association between various metabolites and SCOBY fermentation, key metabolic pathways were mapped and are presented in Figure 8B.

As shown in Figure 8B, the substances in red (green) were the up (down) regulation differential metabolites annotated to the samples, and the substances in black were the intermediates of the metabolic pathways. The result revealed that sucrose and other glycogen were first decomposed into glucose by glycolysis (EMP), and glucose was decomposed into glucose 6-phosphate by the action of hexokinase. Glucose 6-phosphate is an important metabolic substrate in the fermentation process of kombucha, which is closely related to the production of pyruvate and can be isomerized into glucose 1-phosphate by phosphoglucomutase [38]. Pyruvate is an important metabolic centerpiece that produces desulfobiotin, *L*-aspartic acid, and 2-oxoisovalerate, and could synthesize acetyl cofactor by pyruvate dehydrogenase to enter the tricarboxylic acid cycle (TCA cycle) (Figure 8B). The TCA cycle, also known as the citric acid cycle, is the center of carbohydrate, lipid, and amino acid metabolism, which could also generate three important organic acid metabolites including citric acid, α-ketoglutarate, and malic acid [23]. Additionally, glucose 1-phosphate is closely related to the synthesis of glucuronic acid, gluconic acid, and dehydroascorbic acid (Figure 8B). These organic acids are well known metabolites that contribute to the sour taste and improve the flavor of kombucha beverages [18,21]. In addition, glutamate is another important metabolic substrate contained in kombucha, which is metabolized by the amino acid to produce 2-acetamido-5-oxovalerate, *L*-glutamine, oxidized glutathione, and α-ketoglutarate (Figure 8B). Among these metabolites, α-ketoglutarate could also be produced by the metabolism of glucose 1-phosphate, which then participates in the TCA cycle (Figure 8B). Overall, glucose 6-phosphate, pyruvate and glutamate revealed to be the most concerned metabolic components during kombucha fermentation, as they were potential precursors of many flavor-related metabolites and were expected to play an important role in the development of high-quality kombucha products.

## 4. Conclusions

This study demonstrates that the initial sugar concentration affects the sensory attributes, microbial composition, and metabolic profile of RAPT kombucha. Among the four samples tested, the kombucha with 78 g/L sucrose (S4) presented the most favorable sensory qualities, characterized by higher intensities of fruity, alcoholic, and fatty aromas, and received the highest overall liking score. The optimal sugar level promoted the growth of specific microbial genera (*Komagataeibacter*, *Brettanomyces*) and enhanced the biosynthesis of key volatile and non-volatile metabolites, including aroma-active compounds and health-beneficial organic acids. Integrated metagenomic and metabolomic analyses revealed that the pathways related to carbohydrate and amino acid metabolism were highly active under this sugar condition, leading to improved flavor and potential bioactivity. These findings offer valuable insights for better understanding the importance and mechanism of the initial sugar concentration on developing high-quality kombucha beverages.

## Figures and Tables

**Table 1:** Volatile compounds with OAV ≥ 1 in samples fermented on different substrates.

			OAV ^a^			
No.	Compounds	Threshold (μg/kg)	S1	S2	S3	S4
C2	Linalool	0.22	nd	371.91	80.82	41.95
C4	Eucalyptol	1.1	nd	4.06	nd	nd
C11	Geraniol	1.1	nd	3.51	5.98	nd
A1	Ethyl acetate	5	nd	nd	4.06	3.15
B8	Butanoic acid	0.063	nd	127.62	nd	nd
G3	beta-Damascenone	0.002	nd	nd	nd	270.42
E3	4-Ethylphenol	13	nd	9.64	nd	1.32
F6	2,4-Decadienal	0.027	nd	nd	nd	30

^a^ Odor Activity Value (OAV): OAV = Wi/DT; Wi represents the mass concentration of volatile compound in the sample (μg/kg); DT represents the detection threshold of volatile components in water (μg/kg); nd: Not detected.

## Data Availability

The original contributions presented in this study are included in the article/Supplementary Materials. Further inquiries can be directed to the corresponding author.

## Supplementary Materials

The following supporting information can be downloaded at: https://www.mdpi.com/article/10.3390/foods14183216/s1, Table S1: Box-Behnken response surface experimental design and results; Table S2: Results of variance analysis of quadratic model; Table S3: Identification and concentration of volatile compounds contained in kombucha samples; Table S4: Microbial counts of four kombucha samples on the sixth day of fermentation; Figure S1: Analysis of metabolites PCA and PLS-DA in kombucha; Table S5: Details of Difference Components.
