# Supplementary material for: Initial Sugar Concentration on Sensory Characteristics of Raw Pu-Erh Tea Kombucha and Multi-Omics Analysis of the Fermentation Process Under Optimal Sugar Concentration
Source: Foods. 2025 Sep 16;14(18):3216. doi: 10.3390/foods14183216 (PMC12469587; doi:10.3390/foods14183216)
Supplement: Supplementary file 1 [file foods-14-03216-s001.zip › foods-3835713-supplementary.pdf]

Table S1 Box-Behnken response surface experimental design and results

No.	A: Sucrose (%)	B: Tea (%)	C: Fermentation time (d)	Sensory rating
1	9(1)	0.8(0)	7(1)	6.65
2	8(0)	0.8(0)	6(0)	7.59
3	7(-1)	0.8(0)	5(-1)	6.96
4	8(0)	0.8(0)	6(0)	7.62
5	9(1)	0.9(1)	6(0)	6.61
6	7(-1)	0.9(1)	6(0)	6.92
7	7(-1)	0.7(-1)	6(0)	6.93
8	8(0)	0.7(-1)	7(1)	6.72
9	9(1)	0.7(-1)	6(0)	6.65
10	8(0)	0.9(1)	5(-1)	6.81
11	7(-1)	0.8(0)	7(1)	7.01
12	8(0)	0.7(-1)	5(-1)	6.76
13	8(0)	0.9(1)	7(1)	6.87
14	8(0)	0.8(0)	6(0)	7.72
15	9(1)	0.8(0)	5(-1)	6.62
16	8(0)	0.8(0)	6(0)	7.65
17	8(0)	0.8(0)	6(0)	7.69

Final Equation in Terms of Coded Factors: $Y = 7.65 - 0.16*A + 0.0190*B - 0.013*C – 0.0075*AB - 0.005*AC + 0.025*BC - 0.43*A^2 - 0.45*B^2 – 42*C^2$; Response surface optimization solution: Sucrose: 7.8%, Tea: 0.8%, Fermentation time: 6 d. Desirability: 0.955.

Table S2 Results of variance analysis of quadratic model

Source	Sum of squares	df	Mean Square	F Value	*p*-value（Prob>F）	level of significance
Model	2.84	9	0.32	106.60	< 0.0001	***
A	0.21	1	0.21	70.36	< 0.0001	***
B	0.003	1	0.003	0.95	0.3619	***
C	0.001	1	0.001	0.42	0.5363	
AB	2.250E-004	1	2.250E-004	0.076	0.7906	
AC	1.000E-004	1	1.000E-004	0.034	0.8593	
BC	2.500E-003	1	2.500E-003	0.85	0.3884	
A^2	0.77	1	0.77	261.19	< 0.0001	***
B^2	0.85	1	0.85	286.16	< 0.0001	***
C^2	0.73	1	0.73	246.17	< 0.0001	***
Residual	0.021	7	0.003			
Lack of Fit	9.775E-003	3	0.003	1.19	0.4184	not significant
Pure Error	0.011	4	0.003			
Cor Total	2.86	16				
R^2=0.9928		CV=1.52%				R_{Adj}^2=0.9834

*$p < 0.05$, **$p < 0.01$, ***$p < 0.001$

Table S3 Identification and concentration of volatile compounds contained in kombucha samples.

			RI		Concentration (μg/kg)					
No.	Name	CAS	Experiment	Reference	S1	S2	S3	S4	Odor	IM
esters										
A1	Ethyl Acetate	141-78-6	890	894	nd	nd	20.28±1.61[b]	15.77±1.71[a]	fruity, sweet	MS, RI
A2	Butyl butyrate	109-21-7	1879	N	nd	nd	nd	0.66±0.06[b]	fruity, sweet	MS

A3	Ethyl caprylate	106-32-1	1435	1445	nd	1.22±0.09[c]	nd	14.27±0.28[a]	fruity, sweet, fatty	MS, RI
A4	Ethyl caprate	110-38-3	1644	1645	nd	nd	nd	1.13±0.12[a]	fruity, sweet, oily	MS, RI
A5	Ethyl 2-methylbutyrate	7452-79-1	1052	1063	nd	0.91±0.06[b]	13.08±1.24[a]	nd	fruity, sweet	MS, RI
A6	Ethyl Hexanoate	123-66-0	1234	1244	nd	0.46±0.23	nd	nd	fruity, sweet	MS, RI
A7	Dibutyl phthalate	84-74-2	2649	N	0.79±0.02[c]	8.28±1.19[b]	nd	10.90±0.18[a]	faint odor	MS
A8	Methyl salicylate	119-36-8	1793	1770	nd	7.99±0.25[a]	nd	1.18±0.14[b]	wintergreen, mint	MS, RI
A9	Phenethyl acetate	103-45-7	1827	1827	nd	1.20±0.06	nd	nd	rose, sweet, honey	MS, RI
A10	2(3H)-Furanone	104-61-0	2055	2008	nd	nd	nd	0.70±0.04	coconut, creamy, sweet, buttery, oily	MS, RI
A11	Dihydroactinolide	15356-74-8	2381	N	nd	10.66±0.52[a]	nd	4.36±1.02[b]	apricot, fruity, woody	MS
acids										
B1	Acetic acid	64-19-7	1462	1427	2.55±0.19[d]	32.40±1.08[c]	210.44±2.18[a]	44.53±2.84[b]	pungent, vinegar	MS, RI
B2	Octanoic acid	124-07-2	2061	2039	0.74±0.14[d]	1.38±0.46[c]	16.42±0.84[b]	49.12±1.20[a]	fatty, cheesey	MS, RI
B3	Nonanoic acid	112-05-0	2167	2192	0.50±0.06[d]	1.89±0.09[c]	4.86±0.63[b]	8.00±1.27[a]	cheesey, fatty, sweet	MS, RI

B4	Heptanoic acid	111-14-8	1954	1950	nd	nd	nd	0.74±0.09	cheesy, sour, sweet	MS, RI
B5	n-Decanoic acid	334-48-5	2274	2265	2.76±0.88[d]	63.15±5.40[b]	40.58±2.71[c]	105.53±8.70[a]	sour, fatty, citrus	MS, RI
B6	Dodecanoic acid	143-07-7	2483	2503	nd	6.84±0.32[c]	18.05±1.96[a]	9.12±1.54[b]	fatty, coconut bay oil	MS, RI
B7	Propanoic acid	79-31-2	1571	1544	nd	1.39±0.58[a]	nd	0.78±0.06[b]	acidic, buttery, cheesy	MS, RI
B8	Butanoic acid	503-74-2	1672	1644	nd	8.04±0.41	nd	nd	cheesey, sour	MS, RI
B9	Benzeneacetic acid	103-82-2	2578	2569	nd	nd	nd	2.49±0.15	sweet, floral, sour	MS, RI
alcohols										
C1	Ethanol	64-17-5	932	939	nd	3.65±0.24[c]	38.54±3.18[a]	22.88±1.47[b]	alcoholic	MS, RI
C2	Linalool	78-70-6	1550	1544	nd	81.82±4.47[a]	17.78±2.83[b]	9.23±1.71[c]	citrus, woody, sweet, rosey	MS, RI
C3	Phenylethyl Alcohol	1960/12/8	1927	1872	1.18±0.10[e]	34.43±4.63[b]	131.50±3.48[a]	22.34±1.75[c]	floral, rose	MS, RI
C4	Eucalyptol	470-82-6	1205	1220	nd	4.51±0.24	nd	nd	herbal	MS, RI
C5	2-Furanmethanol	5989-33-3	1444	1418	nd	10.38±0.80	nd	nd	sweet, floral	MS, RI
C6	Terpinen-4-ol	562-74-3	1607	1617	0.63±0.12[c]	3.42±0.34[a]	nd	0.83±0.08[b]	woody, musty, sweet	MS, RI

C7	Geraniol	106-24-1	1847	1856	nd	3.86±1.05[b]	6.58±0.82[a]	nd	sweet, floral, fruity, rose, citrus	MS, RI
C8	Benzyl alcohol	100-51-6	1887	1896	nd	6.07±0.76	nd	nd	floral, rose, phenolic	MS, RI
C9	2-Heptanol	543-49-7	1316	N	nd	1.62±0.09	nd	nd	sweet, floral, fruity	MS
C10	α-Terpineol	98-55-5	1700	1718	nd	49.59±6.70[a]	nd	13.71±0.40[b]	citrus, woody, floral	MS, RI
C11	2,6,10-Dodecatrien-1-ol	4602-84-0	2356	2356	nd	2.74±0.14[a]	nd	nd	floral	MS, RI
C12	3-Methyl-1-butanol	123-51-3	1205	1206	0.58±0.04	nd	nd	nd	fusel, fruity, banana, alcoholic	MS, RI
C13	1-Pentanol	71-41-0	1206	1244	nd	nd	nd	1.97±0.13		MS, RI
C14	7-Octen-2-ol	543-39-5	1610	N	nd	nd	nd	0.81±0.04	floral, lavender, citrus	MS
C15	trans-Linalool oxide (furanoid)	34995-77-2	1446	1475	nd	nd	nd	2.40±0.09	floral	MS, RI
C16	5,7-Octadien-2-ol	5986-38-9	1653	N	nd	nd	nd	2.48±0.18	citrus, sweet	MS
terpenes										
D1	D-Limonene	5989-27-5	1201	N	0.30±0.02[d]	3.86±0.30[a]	0.82±0.08[b]	0.59±0.03[c]	sweet, orange, citrus	MS

D2	γ-Terpinene	99-85-4	1245	1254	nd	0.87±0.05	nd	nd	citrus, woody, oily, herbal	MS, RI
D3	Cyclohexene	586-62-9	1283	1280	nd	2.24±0.39[a]	0.84±0.14[c]	1.49±0.05[b]	fresh, woody, sweet, citrus	MS, RI
D4	Styrene	100-42-5	1267	1267	nd	nd	0.28±0.04[b]	0.61±0.03[a]	sweet, floral	MS, RI
phenols										
E1	2,4-Di-tert-butylpheno	96-76-4	2331	2315	1.57±0.21[d]	5.71±0.88[c]	25.05±1.37[a]	7.91±1.38[b]	phenolic	MS, RI
E2	4-Ethyl-2-methoxyphenol	2785-89-9	2043	2058	0.14±0.01[c]	63.05±3.63[a]	nd	3.63±0.26[b]	smoky, bacon, phenolic	MS, RI
E3	4-Ethylphenol	123-07-9	2188	N	nd	125.24±11.36[a]	nd	17.21±2.26[b]	phenolic, castoreum	MS
E4	2-methoxy-4-propyl-Phenol	2785-87-7	2122	N	nd	0.29±0.03	nd	nd	phenolic	MS
E5	2-Methoxy-4-vinylphenol	7786-61-0	2212	2180	nd	1.68±0.10	nd	nd	Phenolic, sweet	MS, RI
E6	2-Ethylphenol	90-00-6	2188	N	0.39±0.07	nd	nd	nd	phenolic	MS
aldehydes										
F1	Benzaldehyde	100-52-7	1541	1530	0.19±0.01[d]	1.93±0.12[c]	9.74±1.27[a]	6.76±0.12[b]	almonds	MS, RI
F2	Benzeneacetaldehyde	122-78-1	1662	1662	nd	nd	0.67±0.08	nd	sweet, floral	MS, RI
F3	2,4-Dimethylbenzaldehyde	15764-16-6	1831	N	2.89±0.02[b]	4.25±0.36[a]	nd	nd	cherry, almond, vanilla	MS

F4	Hexanal	66-25-1	1091	1098	nd	nd	nd	0.48±0.03	pungent	MS, RI
F5	3-Methylbenzaldehyde	620-23-5	1672	1630	nd	nd	nd	5.79±0.28	sweet, fruity, phenolic	MS, RI
F6	2,4-Decadienal	25152-84-5	1828	1826	nd	nd	nd	0.81±0.14	nut, oily	MS, RI
F7	Cinnamaldehyde	14371-10-9	2071	N	nd	nd	nd	2.16±0.11	sweet	MS
ketones										
G1	Cyclohexanone	491-07-6	1475	1503	nd	nd	0.48±0.05	nd	peppermint, sweet	MS, RI
G2	2-Nonanone	821-55-6	1393	1392	nd	nd	nd	28.61±1.47	sweet, herbal	MS, RI
G3	beta-Damascenone	23726-93-4	1837	1838	nd	nd	nd	0.54±0.05	sweet, floral	MS, RI
G4	5,9-Undecadien-2-one	3796-70-1	1865	1867	nd	nd	0.4±0.04[a]	0.32±0.03[b]	fruity, woody	MS, RI
Others										
H1	Cedrol	77-53-2	2141	2149	nd	nd	0.48±0.04[b]	2.51±0.34[a]	woody, sweet	MS, RI
H2	dl-Menthol	89-78-1	1646	1652	nd	nd	nd	0.63±0.06	peppermint, woody	MS, RI
H3	Butylated Hydroxytoluene	128-37-0	1922	1920	nd	3.36±0.56[a]	nd	1.77±0.45[b]	mild phenolic, camphor	MS, RI
H4	2H-Pyran-3-ol	39028-58-5	1764	1770	nd	2.36±1.22	nd	nd	woody	MS, RI

H5	Benzothiazole	95-16-9	1977	N	nd	1.17±0.02	nd	nd	coffee, meat, cooked nutty	MS
H6	Benzene	1195-32-0	1444	1452	0.25±0.01	nd	nd	nd	phenolic, spicy, guaiacol	MS, RI
H7	Furan	7416-35-5	1247	N	nd	nd	0.94±0.11[a]	0.59±0.06[b]	sweet, woody, citrus	MS
H8	1,3,6-Octatriene	3338-55-4	1256	1242	nd	nd	nd	0.54±0.08	sweet, floral	MS, RI

Values correspond to mean values ± standard deviation (SD) of three replicates. nd: not detect. Means, followed by the lowercase letters (a-d) in a line are significantly different ($p < 0.05$) by Duncan's multivariate test (DMRT). IM (Identification method): MS means identification by comparison with the NIST 20 mass spectral database; RI means confirmed by comparison of the retention index with reference standards (https://webbook.nist.gov/). S1: SCOBY fermented with 8% sugar solution that free of tea materials for 6 days, S2: SCOY fermented with 0.8% tea materials that free of sugar for 6 days, S3: SCOBY fermented with 0.8% tea and 3.9% sugar concentration for 6 days, S4: SCOBY fermented with 0.8% tea and 7.8% sugar concentration for 6 days.

Table S4 Microbial counts of four kombucha samples on the sixth day of fermentation

Sample	Plates Counting*		
	Yeast counts (Log CFU/mL)	AAB counts (Log CFU/mL)	Mould counts (Log CFU/mL)
S1	1.13±0.22[d]	0.20±0.10[d]	0
S2	1.76±0.34[c]	0.60±0.10[c]	0
S3	5.84±0.46[b]	6.91±0.31[b]	0
S4	6.18±0.32[a]	7.13±0.41[a]	0

* The microbial plate counting methodology: Kombucha samples were serially diluted in sterile 0.85% saline using the decimal dilution technique, covering a dilution range from 10-1 to 10-8. Yeast, acetic acid bacteria, and mold were counted using YPD, GYC, and PDA plates, respectively. Plates were incubated upside-down at 30 °C for 2–3 days until discrete colonies were observed. Colony counts were performed only on plates containing 30–300 colonies. The number of colony-forming units per

millilitre (CFU/mL) was calculated based on colony counts, dilution factor, and plated volume. If colony counts from two successive dilutions fell within the countable range, the average value was taken. All counts were expressed as log CFU/mL and averaged over three independent replicates; (a-d) in a line are significantly different ($p < 0.05$) by Duncan's multivariate test (DMRT).

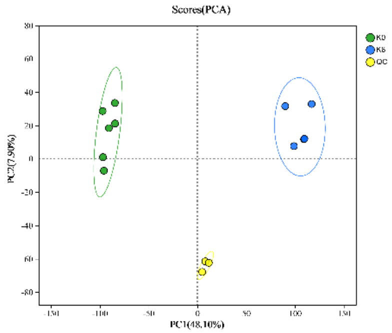

(A)

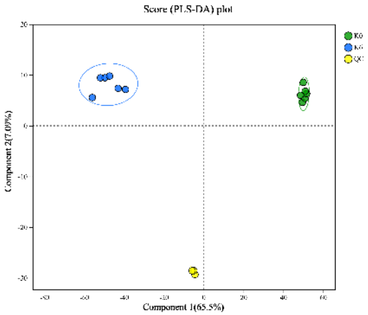

(B)

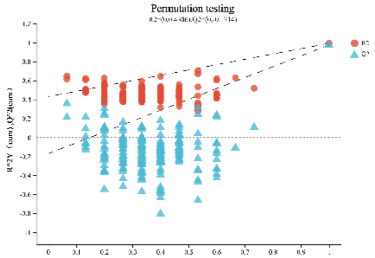

(C)

Figure S1 Analysis of metabolites PCA and PLS-DA in kombucha. (A) PCA analysis chart; (B) PLS-DA analysis chart; (C) PLS-DA permutation test diagram.

K0: unfermented S4; K6: SCOBY fermented for 6 days with S4 used as substrate.

Table S5 Details of Difference Components

ID	Metabolite	Regulate[1]	Model[2]	CAS ID	VIP[3]	FC(K6/K0)[4]	P_value[5]
1	Kaempferol 3-alpha-L-arabinofuranoside	up	pos	5041-67-8	1.7644	1.1947	3.30E-09
2	Sambubiose	down	pos	26388-68-1	1.4837	0.8817	2.12E-08
3	Fraxetin	up	pos	574-84-5	1.8231	1.2495	3.72E-09
4	16-B1-phytoprostane	down	pos	-	1.5319	0.8637	4.87E-14
5	Sphinganine	up	pos	764-22-7	2.4879	1.4766	3.19E-11
6	N2-Acetylornithine	down	pos	6205/8/9	1.2596	0.9352	3.32E-06
7	Pantothenic acid	up	pos	79-83-4	1.0114	1.0509	1.01E-12
8	5-Hydroxy-3,3',7,8-tetramethoxy-4',5'-methylenedioxyflavone	up	pos	161697-25-2	2.5881	1.4169	7.82E-13
9	Diosbulbinoside F	down	pos	66756-58-9	1.3117	0.9084	5.25E-07
10	Moracin P	down	pos	102841-46-3	1.4635	0.8712	2.66E-07
11	Acuminoside	down	pos	120163-17-9	1.475	0.8986	2.08E-08
12	Gingerglycolipid A	down	pos	145937-22-0	3.2526	0.408	1.14E-17
13	Heteroflavanone C	down	pos	156127-36-5	1.9051	0.8068	2.72E-10
14	Egonol	down	pos	530-22-3	1.3932	0.8877	3.82E-09
15	Theaflagallin	up	pos	102208-15-1	2.1078	1.2871	2.19E-12
16	3-Methylbenzaldehyde	down	pos	620-23-5	2.0053	0.7875	3.11E-16
17	2-Keto-6-aminocaproate	up	pos	-	1.3945	1.0923	8.20E-13
18	1,3,9-Trimethyluric acid	up	pos	7464-93-9	3.0371	1.7469	1.53E-15
19	Indoleacrylic acid	down	pos	29953-71-7	2.4776	0.7193	1.58E-07
20	Decarbamoylneosaxitoxin	down	pos	68683-58-9	1.1344	0.9315	3.44E-08
21	4-Hydroxy-3-methoxy-cinnamoylglycine	up	pos	-	1.2154	1.0906	3.26E-11
22	P-Coumaraldehyde	down	pos	20711-53-9;2538-87-6	2.3303	0.7505	1.31E-08

23	Ascladiol	up	pos	32013-85-7	1.7346	1.22	2.57E-08
24	Sarmentosin	up	pos	71933-54-5	2.0907	1.3102	1.36E-12
25	D-Gal alpha 1->6D-Gal alpha 1->6D-Glucose	up	pos	13382-86-0	1.0636	1.0493	1.81E-07
26	5-Ethyl-4-methyloxazole	down	pos	29584-92-7	1.0528	0.943	1.44E-05
27	Tiglylglycine	down	pos	35842-45-6	1.1773	0.9403	1.50E-05
28	Osmundalactone	up	pos	69308-39-0	1.6517	1.1614	1.15E-05
29	Pyroglutamic acid	down	pos	98-79-3	1.2153	0.9229	2.91E-05
30	2-Furoic acid	down	pos	88-14-2	1.2464	0.9249	1.85E-06
31	Niacinamide	down	pos	98-92-0	1.5185	0.8769	6.80E-10
32	Oxidized Glutathione	down	pos	27025-41-8;15718-51-1	1.7371	0.842	1.59E-06
33	2-Aminoacetophenone	down	pos	613-89-8;551-93-9	1.6891	0.8381	6.98E-07
34	(S)-5'-Deoxy-5'-(methylsulfinyl)adenosine	down	pos	897-42-7	3.1026	0.5665	8.14E-15
35	5-Hydroxy-4-pentenoic acid d-lactone	up	pos	26638-97-1	1.1091	1.0751	1.74E-05
36	Herierin III	up	pos	131123-56-3	2.4259	1.3746	3.54E-07
37	3-Keto-b-D-galactose	up	pos	-	2.7991	1.5301	3.40E-08
38	Erinapyrone A	up	pos	146064-66-6	2.2587	1.4158	1.06E-06
39	(4S,6S)-3,4,5,6-Tetrahydro-4-hydroxy-6-methyl-2H-pyran-2-one	up	pos	33275-54-6	2.6499	1.4827	2.59E-14
40	L-Norleucine	up	pos	327-57-1	2.2396	1.2703	5.34E-09
41	(2E)-Decenoyl-ACP	down	pos	52-52-8	1.3001	0.9291	1.34E-10
42	2-Keto-6-acetamidocaproate	up	pos	-	2.3076	1.3225	2.68E-14
43	Homomangiferin	up	pos	21794-66-1	1.7396	1.1642	1.80E-10
44	6-Gingerol	up	pos	58253-27-3;23513-14-6	2.5922	1.8876	5.15E-07

45	4-Ethyl-2-methyloxazole	up	pos	53833-20-8	2.3107	1.2998	2.34E-13
46	Isochinomin	up	pos	83118-66-5	2.6655	1.4327	2.98E-13
47	Arecatannin A1	down	pos	-	1.1141	0.9361	1.07E-06
48	Vanillylamine	down	pos	1196-92-5	1.4813	0.85	6.23E-11
49	Vanillactic acid	up	pos	2475-56-1	1.059	1.0788	1.76E-08
50	Dethiobiotin	up	pos	533-48-2	1.932	1.307	8.92E-11
51	6-Hydroxy-1H-indole-3-acetamide	up	pos	192184-73-9	1.7528	1.2393	1.17E-08
52	1,2,3,4-Tetrahydro-1-[1-hydroxy-3-(4-hydroxyphenyl)-2-propenyl]-7-methoxy-2,6-naphthalenediol	down	pos	163811-77-6	1.3696	0.8904	3.68E-10
53	1,3-dihydroxy-N-methylacridone	down	pos	28333-02-0	1.1848	0.9074	6.64E-07
54	Ineketone	down	pos	62574-18-9	1.8746	0.8106	3.39E-09
55	1-Aminocyclopropanecarboxylic Acid	down	pos	22059-21-8	1.3546	0.8978	2.87E-07
56	Glutarate semialdehyde	down	pos	5746/2/1	1.2255	0.9383	2.59E-06
57	Guanine	down	pos	73-40-5	2.2474	0.742	4.56E-11
58	Piperidine	down	pos	110-89-4	2.1031	0.7653	8.12E-08
59	Agnuside	up	pos	11027-63-7	1.0366	1.0787	0.006917
60	L-erythro-5-(1-Hydroxyethyl)-2(5H)-furanone	down	pos	54621-96-4	1.6243	0.8618	4.68E-08
61	Scopoletin	up	pos	92-61-5	1.876	1.2395	3.79E-14
62	Tiglic acid	up	pos	80-59-1	1.4202	1.1596	4.47E-13
63	2,3-Butanediol glucoside	up	pos	146763-54-4	3.7798	5.5125	1.14E-07
64	N2-Succinyl-L-glutamic acid 5-semialdehyde	up	pos	-	2.1021	1.4426	1.26E-09
65	Tryptophol	down	pos	526-55-6	2.3658	0.6451	3.63E-07
66	L-Allothreonine	up	pos	28954-12-3;24830-94-2	1.6855	1.2354	6.95E-10
67	Amaranthin	up	pos	15167-84-7	1.2401	1.0845	1.16E-06

68	Cajanin	up	pos	32884-36-9	1.9487	1.2317	5.19E-07
69	Cyanidin 3-gentiobioside	down	pos	47845-44-3	1.46	0.8964	9.91E-06
70	Linalool oxide D 3-[apiosyl-(1->6)-glucoside]	down	pos	-	1.3701	0.8991	2.40E-10
71	Apigenin 7,4'-dimethyl ether	up	pos	5128-44-9	2.6495	2.1852	1.49E-08
72	Myristic acid	up	pos	544-63-8	1.3555	1.1256	0.0002426
73	16-Hydroxyhexadecanoic acid	up	pos	506-13-8	1.5455	1.1763	3.19E-05
74	Tetrahydrocorticosterone	down	pos	600-63-5;68-42-8	2.1103	0.7242	3.91E-13
75	1,20-Eicosanediol	up	pos	7735-43-5	1.4798	1.169	0.000304
76	Cer(d18:0/16:0)	up	pos	5966-29-0	1.1539	1.1012	0.0001886
77	Sphingosine	up	pos	123-78-4	2.3039	1.547	1.39E-10
78	Capsiamide	up	pos	64317-66-4	1.485	1.1345	4.33E-05
79	Testosterone	up	pos	651-45-6;58-22-0	2.7282	2.4676	8.34E-06
80	Myristoleic acid	up	pos	544-64-9	2.5644	2.0197	1.06E-08
81	19alpha-19-Hydroxy-3,11-dioxo-12-ursen-28-oic acid	down	pos	-	3.2082	0.426	5.52E-14
82	Methyl (3b,11x)-3-Hydroxy-8-oxo-6-eremophilen-12-oate	down	pos	64964-00-7	1.0857	0.9241	1.86E-08
83	Glycinoeclepin A	down	pos	83216-10-8	1.3256	0.8828	5.11E-10
84	Lactucin	up	pos	1891-29-8	2.7405	2.3263	1.93E-08
85	Aflatoxin B2	up	pos	7220-81-7	2.6245	1.9044	4.39E-07
86	(S)-10,16-Dihydroxyhexadecanoic acid	up	pos	69232-67-3	1.6983	1.2671	1.41E-05
87	5-5'-Dehydrodiferulic acid	up	pos	-	1.1673	1.0982	3.21E-06
88	Cynaroside A	up	pos	117804-06-5	3.0089	2.1762	4.40E-16
89	Kaempferide 3-rhamnoside	up	pos	148435-12-5	1.5076	1.147	1.46E-05

90	N-Acetylserotonin	up	pos	1210-83-9	1.1767	1.1015	3.13E-08
91	1,3-Butanediol	down	pos	107-88-0	1.0994	0.9334	8.26E-11
92	(S)-alpha-Terpinyl glucoside	up	pos	114673-99-3	2.6889	1.8852	4.94E-08
93	Daphnoretin	up	pos	2034-69-7	2.4637	1.5092	4.80E-10
94	Homovanillic acid	up	pos	306-08-1	2.2797	1.4232	1.38E-10
95	Caproic acid	up	pos	142-62-1	1.9287	1.2731	3.69E-07
96	Niazimin	down	pos	159768-73-7	2.5184	0.6621	3.35E-09
97	Genkwanin	up	pos	437-64-9	1.5948	1.1642	9.92E-06
98	6''-O-Malonylglycitin	up	pos	137705-39-6	2.422	1.55	1.81E-07
99	Emodin	down	pos	518-82-1	1.8008	0.8376	1.94E-13
100	Apigenin	down	pos	520-36-5	1.1992	0.9126	0.0001464
101	Cyanidin-3,5-diglucoside	down	pos	-	1.6905	0.8561	3.06E-06
102	Cirsilineol	down	pos	41365-32-6	1.5841	0.8759	1.60E-10
103	Valrubicin	down	pos	41744-33-6;56124-62-0	1.8826	0.7973	9.13E-06
104	Tetraphyllin B sulfate	up	pos	85758-30-1	1.4897	1.1567	3.90E-09
105	5-Methyl-2-furancarboxaldehyde	up	pos	620-02-0	1.1797	1.1026	7.97E-07
106	Delphinidin 3,5-diglucoside	down	pos	17670-06-3	1.8058	0.8294	3.11E-06
107	Tetracycline	up	pos	60-54-8	2.6375	1.6928	1.30E-07
108	9-Hydroxy-4-methoxypsoralen 9-glucoside	up	pos	115356-06-4	2.5509	1.5528	2.67E-12
109	Oxytetracycline	down	pos	79-57-2	1.7446	0.8546	1.00E-06
110	Cernuine	down	pos	6880-84-8;480-70-6	1.3495	0.8944	3.21E-05
111	Serotonin	down	pos	50-67-9	2.3249	0.6579	9.29E-09
112	3'-Hydroxygenistein	down	pos	-	1.6588	0.8523	2.14E-07
113	3-Hydroxycarbamazepine	up	pos	68011-67-6	1.6618	1.188	6.03E-09

114	L-Pipecolic acid	up	pos	3105-95-1;535-75-1	1.288	1.0939	1.42E-08
115	Oleoside dimethyl ester	down	pos	30164-95-5	2.1342	0.7347	2.20E-07
116	Gamma-Glutamylvaline	down	pos	2746-34-1	1.3107	0.9012	3.89E-10
117	Calystegine C1	up	pos	156705-04-3	2.1654	1.3	5.22E-14
118	2-Keto-glutaramic acid	up	pos	18465-19-5	2.6721	1.7633	3.25E-14
119	2-Hydroxybutyric acid	up	pos	600-15-7;3347-90-8	1.864	1.2618	2.17E-11
120	L-3-Aminodihydro-2(3H)-furanone	down	pos	-	1.0635	0.9305	1.23E-08
121	Butyric acid	up	pos	107-92-6	1.9139	1.3253	1.57E-11
122	Fagomine	up	pos	53185-12-9	1.5011	1.1139	1.06E-08
123	Isopropylmaleic acid	up	pos	44976-69-4	2.5805	1.4169	1.02E-14
124	Diethanolamine	up	pos	111-42-2	2.6273	1.4744	5.27E-12
125	Aminocaproic acid	down	pos	60-32-2	1.759	0.8466	7.68E-08
126	Norfuraneol	up	pos	19322-27-1	1.3159	1.1019	1.55E-11
127	Adenosine	down	pos	58-61-7	3.0568	0.5768	7.88E-09
128	Succinic acid semialdehyde	up	pos	692-29-5	1.7783	1.236	3.17E-07
129	Cis,cis-Muconic acid	up	pos	1119-72-8	1.2604	1.0891	5.78E-05
130	N-Acetyl-L-glutamate 5-semialdehyde	up	pos	13074-21-0	1.6269	1.1336	3.26E-11
131	4-Hydroxybenzaldehyde	down	pos	123-08-0	1.216	0.9111	1.30E-07
132	2-Methylbenzoic acid	down	pos	118-90-1	1.625	0.8321	1.15E-07
133	N-Acetylmannosamine	up	pos	7772-94-3	1.0057	1.0511	3.42E-11
134	Glycine-betaxanthin	up	pos	-	2.6482	1.5539	4.21E-08
135	4-Trimethylammoniobutanoic acid	down	pos	407-64-7	1.9717	0.8074	7.47E-13
136	Spermidine	up	pos	124-20-9	1.5174	1.1765	1.46E-05
137	4-Hydroxycinnamic acid	down	pos	7400-08-0;501-	1.6774	0.8611	1.98E-07

				98-4;4501-31-9;20649-40-5;501-98-4			
138	D-Apiose	up	pos	639-97-4	1.4573	1.1931	1.60E-05
139	Dodecanoic acid	up	pos	143-07-7;1908-11-8	1.5615	1.1839	0.000353
140	Genistein	down	pos	446-72-0;529-59-9	1.0045	0.9471	1.18E-10
141	Indole-3-Carboxaldehyde	down	pos	487-89-8;87-51-4;58-63-9	2.0773	0.7629	4.99E-10
142	Mesaconic acid	up	pos	498-23-7;498-24-8	2.7159	1.7906	1.34E-11
143	Phenylacetylglutamine	up	pos	28047-15-6	1.7373	1.2482	1.63E-11
144	Phloretin	up	pos	7507-89-3;60-82-2	2.6122	1.6727	1.08E-09
145	Prenol	down	pos	556-82-1	2.0938	0.6745	9.85E-11
146	Raffinose	up	neg	512-69-6	2.2868	1.2918	1.19E-12
147	4'-Methyl-(-)-epigallocatechin 3-(4-methyl-gallate)	up	neg	-	1.9067	1.2308	6.96E-07
148	Neryl arabinofuranosyl-glucoside	down	neg	84534-32-7	1.309	0.9191	1.26E-08
149	Lyoniresinol 9'-sulfate	up	neg	-	3.0783	2.3227	3.88E-09
150	(S)-Nerolidol 3-O-[a-L-rhamnopyranosyl-(1->2)-b-D-glucopyranoside]	down	neg	130466-30-7	3.121	0.4409	1.13E-09
151	Calystegine B2	up	neg	127414-85-1	2.9289	1.7029	4.83E-13
152	4-Acetamidobutanoate	up	neg	3025-96-5	1.9449	1.2361	1.95E-12
153	3'-Ketolactose	up	neg	15990-62-2	1.8011	1.2812	0.0004494

154	Citric Acid	up	neg	77-92-9	1.4941	1.0954	2.25E-15
155	2-Isopropylmalic Acid	up	neg	49601-06-1	1.3944	1.0949	1.39E-10
156	D-1,5-Anhydrofructose	down	neg	75414-43-6	1.9655	0.7988	1.91E-15
157	Xanthotoxol arabinoside	down	neg	160845-06-7	1.3367	0.9084	4.43E-11
158	Epitheaflagallin 3-O-gallate	up	neg	102067-92-5	2.8223	1.8758	4.23E-11
159	Bancroftinone	up	neg	14964-98-8	1.0466	1.0884	2.99E-06
160	3-(3-hydroxyphenyl)propanoic Acid	up	neg	621-54-5	2.0015	1.2983	7.82E-12
161	Corchorifatty acid D	down	neg	-	2.7478	0.5138	1.51E-10
162	4-Caffeoyl-1,5-quinolactone	up	neg	-	1.4112	1.1223	1.93E-09
163	17-Hydroxylinolenic acid	down	neg	-	2.0511	0.7701	8.62E-10
164	D-Gulono-1,4-Lactone	up	neg	-	1.5042	1.1516	4.28E-06
165	L-Glutamine	down	neg	56-85-9;5959-95-5	1.7361	0.826	2.00E-09
166	L-Aspartic acid	down	neg	56-84-8	2.4411	0.6599	6.04E-08
167	L-Asparagine	down	neg	70-47-3	1.9837	0.7523	1.30E-06
168	Glycolaldehyde	down	neg	141-46-8	1.0084	0.9531	3.55E-09
169	Dihydroxyacetone Phosphate Acyl Ester	up	neg	-	1.4236	1.1374	3.86E-06
170	N-trans-p-Coumaroyloctopamine	down	neg	66648-45-1	1.3256	0.9015	2.07E-08
171	Galactinol	down	neg	3687-64-7	1.5584	0.9048	2.37E-06
172	Gluconolactone	up	neg	90-80-2	3.1906	1.8898	1.07E-10
173	Xylobiose	up	neg	6860-47-5	2.5056	1.413	5.60E-13
174	Citramalic Acid	up	neg	2306-22-1;597-44-4	1.9036	1.199	2.53E-14
175	B-D-Glucuronopyranosyl-(1->3)-a-D-galacturonopyranosyl-(1->2)-L-rhamnose	up	neg	-	2.6512	1.4569	8.64E-14
176	11-Deacetylvaltrate 11-(3-hydroxy-3-	down	neg	96681-66-2	2.306	0.7636	7.74E-07

	methylbutanoate)						
177	D-Xylono-1,5-lactone	up	neg	-	1.8038	1.1766	8.98E-10
178	Xanthine	up	neg	69-89-6	2.6292	2.0108	1.64E-06
179	D-glycero-L-galacto-Octulose	up	neg	-	1.6861	1.2472	0.0001577
180	L-Hypoglycin A	up	neg	156-56-9	2.9747	1.7528	2.52E-14
181	2-Oxo-5,11(13)-eudesmadien-12,8-olide	up	neg	68776-46-5	2.2867	1.4485	2.47E-13
182	3-(4-Hydroxyphenyl)lactate	up	neg	306-23-0	1.7234	1.2025	1.16E-09
183	Liquiritigenin	down	neg	578-86-9	1.3859	0.8618	0.0001702
184	Cajanol	down	neg	61020-70-0	1.4134	0.8878	1.10E-11
185	4'-O-Methyl-(-)-epicatechin 3'-O-glucuronide	up	neg	-	1.8339	1.2447	1.95E-06
186	Ethyl 2-furanyl diketone	up	neg	1438-90-0	2.1138	1.3865	3.68E-10
187	(S)-Rutaretin	down	neg	-	1.4394	0.8577	8.50E-09
188	Quercetin 3-(6''-malonyl-glucoside)	down	neg	96862-01-0	1.5009	0.8914	2.83E-08
189	Kuwanon Y	down	neg	105016-28-2	1.6222	0.827	2.70E-05
190	3-Methylellagic acid 8-(3-acetylrhamnoside)	up	neg	-	2.2458	1.5684	2.95E-05
191	Betavulgarin	up	neg	51068-94-1	1.5794	1.181	2.56E-06
192	2-Methylpropyl 3-hydroxy-2-methylidenebutanoate	up	neg	80758-68-5	1.2286	1.1212	1.33E-09
193	Indolelactic acid	up	neg	7417-65-4	2.6016	1.799	2.29E-10
194	(-)-Epiafzelechin	up	neg	24808-04-6	1.4444	1.1445	3.04E-11
195	2-Methoxyestrone 3-glucuronide	down	neg	25577-70-2	2.4638	0.6175	2.21E-08
196	2'',4''-Diacetylafzelin	up	neg	133882-73-2	1.0125	1.0685	5.74E-08
197	9-F1-phytoprostane	up	neg	-	1.7042	1.2438	1.22E-11
198	Fisetin	up	neg	528-48-3	1.0655	1.0631	1.91E-08
199	(9R,10S,12Z)-9,10-Dihydroxy-8-oxo-12-octadecenoic acid	up	neg	142036-13-3	1.3159	1.1232	2.04E-10

200	9,12,13-TriHOME	up	neg	-	1.4911	1.1701	2.61E-10
201	Goshonoside F2	down	neg	90851-25-5	2.9628	0.4772	3.07E-13
202	Cafestol	down	neg	469-83-0	2.2244	0.6707	1.27E-09
203	(2'E,4'Z,7'Z,8E)-Colnelenic acid	down	neg	52591-16-9	2.9765	0.3983	2.41E-12
204	5,8,12-Trihydroxy-9-octadecenoic acid	up	neg	-	1.3986	1.1538	4.59E-10
205	Undecylenic acid	up	neg	112-38-9	1.0161	1.0928	3.74E-05
206	Phlorizin	down	neg	60-81-1	1.4681	0.8649	1.51E-08
207	2-(1-Pentenyl)furan	up	neg	81677-78-3	2.9126	2.9585	3.05E-11
208	3,4-Di-O-caffeoylquinic acid	up	neg	57378-72-0;14534-61-3	2.8658	1.9865	1.97E-08
209	Aflatoxin P1	up	neg	32215-02-4	2.5644	1.6447	1.04E-11
210	2'-O-Methylisoliquiritigenin	up	neg	112408-67-0	2.5224	1.7214	1.43E-08
211	Irilone	up	neg	41653-81-0	2.4092	1.6363	3.37E-10
212	Tyrosol	up	neg	501-94-0	2.4095	1.6718	6.12E-11
213	Isoneotheaflavin	down	neg	-	1.0289	0.9291	7.38E-09
214	Luteolin 3'-(3''-acetylglucuronide)	down	neg	-	1.1014	0.9258	1.69E-09
215	3,8-Dihydroxy-1-methylanthraquinone-2-carboxylic acid	up	neg	69119-31-9	1.6073	1.1993	3.57E-09
216	Wedelolactone	up	neg	524-12-9	2.1837	1.5328	4.91E-08
217	Mycorradicin	down	neg	160162-46-9	2.0242	0.7442	7.09E-09
218	Laccaic acid D	up	neg	18499-84-8	2.439	1.645	3.51E-11
219	Sakuranetin	up	neg	2957-21-3	1.5075	1.1862	7.82E-06
220	Gibberellin A34-catabolite	down	neg	-	1.3043	0.8769	4.11E-06
221	Biochanin A 7-(6-malonylglucoside)	up	neg	34232-17-2	2.7375	1.499	4.03E-13
222	Na-p-Hydroxycoumaroyltryptophan	up	neg	-	2.415	1.5442	7.04E-11
223	Ketoleucine	up	neg	816-66-0	1.1907	1.1123	1.77E-08

224	4-Methylumbelliferone glucuronide	up	neg	6160-80-1	1.5078	1.1796	2.91E-05
225	Scutellarein 6-xyloside	up	neg	65876-68-8	2.2503	1.3646	5.17E-12
226	Glutamine betaine	up	neg	-	1.1045	1.0757	1.50E-06
227	3-Isopropylmalic acid	up	neg	126576-14-5	1.5162	1.1456	1.06E-09
228	Monotropein	up	neg	5945-50-6	1.469	1.1635	7.99E-07
229	6,7-Dimethyl-8-(1-D-ribityl)lumazine	down	neg	5118-16-1;2535-20-8	1.2051	0.9193	3.14E-10
230	Salvianolic acid G	up	neg	-	2.1094	1.3346	4.08E-11
231	3-O-alpha-L-Arabinopyranosylproanthocyanidin A5'	up	neg	-	2.8305	1.7146	2.18E-12
232	(-)-Epigallocatechin 3'-glucuronide	up	neg	-	2.0216	1.2642	4.18E-09
233	Vanillylmandelic Acid	down	neg	13244-77-4;55-10-7	1.8644	0.748	9.84E-09
234	5-(3-Pyridyl)-2-hydroxytetrahydrofuran	up	neg	53798-73-5	1.0123	1.0827	1.56E-08
235	L-Tryptophan	down	neg	73-22-3	2.2752	0.7043	2.21E-08
236	Tricholomic acid	up	neg	2644-49-7	3.6084	6.932	3.27E-17
237	Loxoprofen	up	neg	80382-23-6	2.5578	1.6225	7.47E-12
238	(S)-Multifidol 2-[apiosyl-(1->6)-glucoside]	up	neg	467437-62-3	2.0097	1.3133	7.39E-10
239	Catechin 7-glucoside	down	neg	65597-47-9	1.2028	0.9137	6.27E-11
240	(-)-Epigallocatechin 7-glucuronide	up	neg	-	1.6982	1.2138	3.54E-08
241	2'-Hydroxynicotine	up	neg	1824020-12-3	2.3255	1.4728	3.02E-13
242	L-Phenylalanine	down	neg	63-91-2	2.0297	0.7735	4.54E-10
243	Furfuryl acetate	up	neg	623-17-6	1.1175	1.0851	3.40E-12
244	Egonol gentiobioside	down	neg	-	1.1361	0.9351	5.82E-06
245	3-Hydroxy-3-methylglutarate	up	neg	503-49-1	1.7289	1.1651	1.51E-13
246	A-L-Arabinofuranosyl-(1->3)-[a-L-	up	neg	-	1.3527	1.1039	2.24E-08

	arabinofuranosyl-(1r5)]-L-arabinose						
247	L-4-Hydroxyglutamate semialdehyde	down	neg	-	1.6799	0.8548	7.23E-09
248	3h-Sialic acid	down	neg	-	1.0792	0.9245	4.75E-05
249	Oxoglutaric acid	up	neg	328-50-7	2.7939	1.6373	1.25E-15
250	Glucaric acid	up	neg	87-73-0	3.2231	1.8428	3.18E-11
251	Pentose	down	neg	9000-69-5	1.2468	0.9089	1.97E-11
252	Chrysoobtusin	down	neg	70588-06-6	1.2565	0.9259	1.43E-06
253	Methylmalonic acid	up	neg	516-05-2	1.18	1.0788	1.55E-08
254	D-Arabinono-1,4-lactone	up	neg	2782/9/4	2.3011	1.3151	9.60E-14
255	(S)-3-Sulfonatolactate	up	neg	-	1.1532	1.1047	4.39E-09
256	Phosphinomethylisomalate	up	neg	-	1.7659	1.2289	9.98E-07
257	D-Glucuronic acid	up	neg	70021-34-0;6556-12-3;32449-92-6	2.6132	1.4293	4.45E-14
258	D-Ribose	up	neg	613-83-2;50-69-1	2.0216	1.2061	1.00E-07
259	Dehydroascorbic acid	up	neg	490-83-5	2.3234	1.3228	9.48E-18
260	Inulobiose	down	neg	470-58-6	1.2197	0.9359	1.58E-06

Regulate[1]: Differential metabolite up-regulation (up) or down-regulation (down); Model[2]: the differential metabolite is in cationic mode (pos) or anionic mode (neg); VIP[3]: Significance of Variables Obtained by OPLS-DA Modeling; FC(K6/K0)[4]: Substance multiplicity relationship between the two groups; P_value[5]: The p-value obtained from Student's t test. K0: unfermented S4; K6: SCOBY fermented for 6 days with S4 used as substrate.
